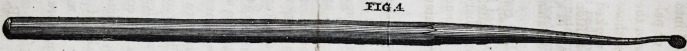# Crystalline Gold, Its Varieties, Properties and Use

**Published:** 1855-04

**Authors:** Wm. H. Dwinelle


### ARTICLE XI.

*Crystalline Gold, its Varieties, Properties and Use.* By WM. H. DWINELLE, M. D., D. D. S.

GOLD is indispensable to commerce; is one of the greatest incentives to action and to enterprize; is an emblem of royalty and power; is universally coveted, and is the father of many a crime; it enriches our language with some of its most glowing adjectives, swell the remedial catalogue of medicines, and finds valuable uses throughout the whole range of the arts and sciences.

But nowhere, and in no way, is it at the same time such a needful luxury and a continual comfort as in our Art. Here it realizes its highest end, and becomes exalted in its power to save, while it intimately mingles, and becomes permanently associated with living organs. Nature herself seems to have kindred feelings towards it, and permits it in such close proximity to her vital organs, that it sometimes nearly reposes upon blood-vessels, whose pulsations almost beat against it. At other times it harmlessly forms an arch under which delicately tissued nerves send many a subtle current of feeling. Gold ever has been, and probably ever will be, regarded as the best material for plugging and arresting the progress of caries in teeth. Its capability of being attenuated into foil or finely divided crystals, and these afterwards of being consolidated into a united mass, its density when properly worked, its indestructibility, its color and non-corrosive quality, renders it superior to any other material for the purpose of filling cavities of the teeth, where it will resist with impunity the ever varying and vitiating fluids of the mouth.

The history, character and uses of gold foil, are too well known to our profession to require any detailed description, at this time. It is well known that its desirable qualities for our purpose, depend entirely upon its purity, and the manner in which it is manufactured—purity, however, always being an essential element of its goodness. It is unnecessary to refer to the excellences of the article for the purpose for which it is intended. In the hands of the skillful operator, it is daily restoring the perishing organs of the mouth to permanent health and usefulness.

It has done more towards elevating our profession to its present high position that any other cause. I have no inclination, nor intention, of speaking disparagingly of it. All that could be done with it has been long since accomplished, and that is much, and far more than the fathers in our profession could have anticipated. In comparing it with crystal gold, the objections to it must only be of a negative character. Slightly changing a familiar declaration, I may say, "not that I prize the one less, but the other more!" It is not that foil does not accomplish much, but that crystal gold accomplishes more. Foil, by skillful manipulation, may be made comparatively solid. Crystal gold, by the same means, may be made absolutely so, becoming more dense even than coin. Foil is sometimes *com-*

*paratively integrated;* that is, each part is resolved into the unity of the whole. Crystal gold *can always be so completely integrated,* as to endure every test that coined gold can withstand.

When the best foil stopping is removed from the tooth and crushed by a heavy lateral or sliding pressure, it is partially separated into irregular and laminated forms. This is very distinctly seen whenever the stopping is broken up in the mouth. The order, or manner of its disintegration, is as follows: The fluids of the mouth penetrate through and beneath the lamina, or around and about the borders of the pellets, gradually upheaving and disrupting the integrity of the whole.

Crystal gold stoppings, when properly made, have the quality of oneness so complete, each ultimate particle being so thoroughly united and integrated into the whole, that it is no more likely to break up or separate from itself than any other equal bulk of pure solid gold. In case an angle or portion of it should be broken off, it would be the same as though an equal amount were separated from any other piece of highly tempered gold. If desirable, the lost portion can be replaced; if not, it may be filed and burnished down to a new surface, and left, without in the least endangering the remaining portion of the stopping. It is a fact well known to the profession, that when foil is broken up into angles and formed into coils or pellets, it thereby often becomes so tempered, so hard and harsh, that it is worked only with great difficulty; the laminated structure of the gold is so bent and angulated into thousands of arches, in archings and bracings, as to resist a large force applied to the top of the mass, without materially breaking up the structure at the bottom. To obviate this, it is necessary to introduce the gold in smaller masses of less degree of density, and to forcibly work them together with the most delicate pointed instruments.

Stoppings made by our best artists, when placed under the microscope, will be found to have their inner surface entirely traced over with irregular, sinuous, concave groovings, extending from the centre to its borders—the remains of the angular presentation of the foil to the surface of the cavity. When these semi-tubular groovings are too small to absorb the fluids of the mouth, their presence is not injurious; but when larger, the ultimate disruption of the stopping is inevitable. Crystal gold has a peculiar quality which permits it to be introduced into the cavity in comparatively large masses, which may afterwards be thoroughly condensed in their place. When properly worked, it gives the sharpest impression of all the irregularities and most delicate markings of the inner surface of the cavity, and absolutely insinuates itself into the texture of the tooth. This fact is easily demonstrated by the microscope.

In using crystal gold, it will, however, be necessary in order to secure the highest degree of success, to take into consideration the nature and character of the article to be used; wherein it differs from foil; and the peculiar manner of treatment it requires; for, in many respects, the system of manipulation which would secure the best foil stopping, would utterly fail of success when applied to crystal gold.

Crystal gold, as its name indicates, is made up of a combination of crystals of pure gold, so interlaced and interwoven that upon being submitted to pressure, it readily welds into a solid mass.

By leaving its upper surface rough in the act of filling, layer may be built upon layer, until any desirable thickness is attained.

As the arrangement of the crystals of the gold here mentioned, are in the most perfect condition for complete consolidation, it should be the study of the operator to avoid breaking up the formation, any more than is absolutely necessary, and, so far as possible, to give a *direct* pressure upon the gold, especially in the early part of the operation—bearing in mind that all wedge-shaped instruments should be dispensed with, in *introducing* the gold into the cavity, till after the gold is fixed to its place and partly condensed.

The character of this gold so differs from that of foil, that even when introduced in masses, upon direct pressure being applied, it so yields upon itself, that it may be readily carried to the remotest point of the cavity, and there consolidated against its wall.

Unlike the smooth surface of foil, it presents myriads of angles to the opposing dentine, and insinuating itself into its texture; for this reason, and also that the gold becomes constantly and absolutely solid as the operation advances, it is not necessary, as much as heretofore, to *form* cavities to ensure the retention of the fillings.

It will be well for those who have not had experience in the use of crystal gold, to confine their operations for a while, to that class of cavities which can be directly and easily approached: after they have acquired practice in the use of the material, the instruments and their management, lateral and more difficult cavities may be attempted.

In case the gold should become wet during the operation of filling, thoroughly condense that already in the cavity, then burnish its surface and let the patient rest. In resuming the operation, brush out any crumbles of gold that may remain, dry the cavity and surface of the filling with coils of tissue or bibulous paper; then, with a sharp-pointed condensing instrument, stipple over the entire face of the stopping, taking advantage of all irregularities or undercuts, again dry the gold with paper, and proceed as before the accident—only taking care to work the first succeeding layer *of gold* thoroughly into the texture of that which underlies it.

In case the gold loses its adhesive quality by dampness or exposure, simply drying it over a spirit lamp, will restore it to its original condition.

It is now more than two years since I commenced the use of crystal gold, manufactured by A. J. Watts & Co., of Utica. Since April last I have used it exclusively in my practice. Almost daily I meet with stoppings of this preparation of gold put into the teeth of my patients from the time of my first using it, and so far from my experience or observation having the effect of impairing my confidence in the new material, I daily congratulate myself upon being able to produce results which never could have been accomplished with foil.

While all ordinary cavities may be filled with crystal gold, in a far superior manner to what it is possible to stop them with foil, it especially commends itself to an important class of valuable teeth, hitherto confessedly beyond the reach of foil or gold—in any other form—to save. I refer, 1st, to a large large class of frail teeth, whose walls are so thin and weak, as to be unable to resist the pressure of filling, and the lateral bearing of the plug necessary to its retention. And 2nd, to a class of teeth always deemed beyond the reach of art to save, namely, teeth without crowns, or of which but a fraction remains.

Hitherto all of this first class of teeth, if treated at all, were filled with indifferent or perishable materials. The second class were almost invariably suffered to be lost. When properly prepared, crystal gold may be effectually consolidated into cavities of the frailest teeth, without danger of fracture or breakage, in such a manner as to reproduce the entire lost substance of the organs, and ensure the whole against further decay.

It requires for its treatment an entirely different system of manipulation from that of foil. For this reason, and owing to peculiar qualities to which we will again allude, it is not only well adapted to all classes of decayed teeth, but eminently so to the particular class just referred to. The principal reason why a large share of frail teeth are not successfully filled with foil is, that the wedging and lateral pressure, necessary to its retention, and to secure a sufficiently solid plug, inevitably spread apart and break up their frail walls; so that often, when a large stopping of foil has been introduced into such a tooth, its walls are then more likely than ever to break, from the fact that an undue lateral pressure gives the foil a larger leverage upon them, especially at the orifice, maintaining a continual tendency to spread and separate them from each other.

These remarks apply with especial force to bicuspids and molars which have lost the anterior and posterior parts of their crowns, leaving only the labial and lingual walls. Crystal gold requires but little lateral pressure in its condensation. The very nature of its structure forbids it. Wedge-shaped instruments, especially if used before the gold is partly condensed, only break up its texture, and subject it to waste. It must be introduced in masses that are nearly covered by the presenting surface of the instrument used: with these it should be carried to its place, and there condensed, as far as possible: it should then be followed by smaller instruments, and by direct or perpendicular pressure, consolidated. That portion of gold alone, which is immediately under the instrument, receives the pressure, which is not taken up and distributed throughout the whole plug as with foil—hence the several portions of gold, as they are successively carried to their place, require but little of the lateral pressure necessary for foil.

As layer upon layer succeeds each other, they become absolutely solid and completely integrated into the general mass; so that when the stopping is finished, its quality of unity is as complete as though it had been cast from the crucible. On account of this peculiar property by taking advantage of the inequalities of the external walls of their cavities, the frailest teeth may often be so overlaid and interlaced with the constantly solidifying gold, as to have their walls effectually bound together, not only securing them their original position and condition, but fortifying them in it.

In these cases the remaining portions of the enameled cusps become *inlaid*, as pearls are inlaid in gold. Frail teeth of this character may not only have their opposite walls banded together by the inequalities of the external opening, but by their internal irregularities, which oftentimes approach the character of *dove-tailings*, as will be seen by figs. A, B, C, D, E, F, and G, hereinafter to be described.

It often occurs to the dentist, while extracting teeth which have only lost their crowns, and even roots of teeth which are well established in their places, that it is a matter of deep regret that such a superior and independent foundation for a tooth—one so pre-eminently beyond the reach of art to supply, should be destroyed, to be replaced, if replaced at all, by a clumsy fixture, covering a large portion of the palate, or, depending upon the adjoining teeth, insuring their ultimate destruction. Fortunately, here again, crystal gold comes to our aid, and enables us, not only to replace and secure any remaining portion of the crown, but in many instances, to reproduce the entire crown itself. More than this, in case of the upper bicuspids, the entire crown has been reproduced in gold, with an enamel cap on the buccal surface, so as in all respects to represent and take the place of the outer cusp. The method of operating on the three last named kinds of teeth, will be mentioned in detail in this treatise.

The frail teeth spoken of will be ranged under the head of Class I. Molars and bicuspids, with parts of, or without crowns, under the head of Class II. Bicuspids, with the gold crowns and artificial cusps, under the head of Class III.

For the benefit of those who are inexperienced in the use of crystal gold, I will undertake to give a more detailed description of the method of operating with it upon all classes of teeth, beginning with simple and ordinary cases, gradually approaching those more complex in their character, and ending with the special classes, arranged above, under the heads of Class I, II, and III.

Before proceeding with a description of the method of operation, let us again consider the character of the material we are about to use, in order to supply ourselves with instruments adapted to its peculiarities, as well as to impress ourselves with the system of manipulation it requires. Foil is *thrust* to its place and worked into its own texture, by a system of *wedging*. Crystal gold *packs;* it is adhesive and semi-plastic. As one layer succeeds another, under the direction of proper instruments, it adheres to and integrates into the preceding layers, as legitimately and completely, as each added portion of clay, under the hands of the artist, becomes resolved into the unity of the whole of the figure before him. So we must have instruments which shall adapt themselves to its singular qualities. The adhesive and plastic nature of the material at once suggests instruments, approaching in character the modeling tools of the sculptor—some form of instrument by which we can take up the plastic material in broad masses, thin layers, attenuated strips or small particles, carry them to their place, and build upon and model, while we continually consolidate the whole.

For the purpose of introducing the crystal gold to its place, and partly consolidating it, instruments that are a kind of *cross* between a plugging and a modeling tool, will be found to answer the above requirement, and to be of indispensable usefulness to our purpose. Their forms may be modified or multiplied, according to the caprice, the genius, or the needs of the operator. Several sizes of each will be required, gradually approximating to the smallest and most delicate.

No. 1 represents a broad-faced instrument, slightly rounded at its edges, bent at an angle, the working end being serrated on all sides. The profile view shows that it is made thin, for the purpose of increasing its delicacy, as well as to admit of its being used for placing gold between approximal and slightly separated surfaces. It is useful for many purposes, but chiefly for carrying masses of crystal gold to their place, and modeling and shaping them into form. Instruments of this character, but of smaller size, are particularly useful in introducing gold into front teeth.

No 1 No 2.

No. 2 is a serrated instrument of the same general character, but broader and thicker, conical in form, and terminating in a point at its apex. It is an exceedingly fine modeling tool, and is especially useful in introducing large masses of gold into cavities in the posterior surfaces.

No 3.

No. 3 is also of the same general character, circular in shape, convex on one side, and flat on the other, invented by Dr. W. M. Hunter, of Cincinnati. In some parts of the operation it is more useful than any other form. By rotating the instrument in the act of filling, the progress of consolidation is greatly facilitated. Its opposite or flat surface, from its concave curve, is very useful in introducing gold into posterior approximal cavities, or in all operations where the dentist must work towards himself.

No 4.

No. 4 is an instrument bent at a slight angle, flat on its upper surface, and terminating in a square point. The under surface is convex, gradually rounding at the sides until it meets the angle of the flat upper surface. Instruments of this character are more useful than others, for the reason that they combine all the advantages of three or four instruments in one, so that we can apply several different kinds of forces, without laying down the instrument. The upper and lower surfaces, the sides, and the ends, furnishing different and practical presentations, all of which are commanded by simply giving the instrument a part of a revolution.

No 5.

No. 5 represents an instrument slightly modified from the former, the chief difference arising from its having a concave groove cut out immediately behind the convex presentation of the under surface, leaving the latter prominent, and giving the instrument a *neck* at this point, enabling the beaded prominence to pass for a short distance into the cavities of posterior approximal presentations, while the curved neck of the instrument avoids the projecting angle of the tooth, which would otherwise interfere. It is very useful also in working the gold into the undercut angles of large molar cavities.

These instruments may be bent to more acute angles or curves, and diminished to the most delicate form with great advantage. With these modifications they are often very serviceable in filling cavities in front teeth.

No 6.

No. 6 is a flat, thin, cone-formed instrument, diverged to a slight angle, and is particularly suitable for introducing gold into the approximal surfaces of front teeth. It is also very useful in securing good joints about the borders of large molar stoppings, the point and edge of the instrument being used for this purpose with great advantage, care being taken during the operation of condensing and finishing the stopping, so to place the point of the instrument upon the filling, as to have a small bordering of gold intervene between itself and the enameled edge of the cavity to prevent fracturing it.

Nº 7.

Nº 8.

No. 7 represents a right and left instrument, with a parabolic, or rather, a wave-line curve, turned at an angle of about 35° from its shaft. Each side is serrated. It is very useful in filling all classes of approximal cavities, and was invented by Prof. R. Arthur, of Philadelphia.

No. 8 is a serrated blade instrument, bent at various angles, and of different degrees of thickness, used principally in filling front and approximal cavities.

Nº 9. Nº 10. Nº 10X.

No. 11. No. 12.

Nos. 9, 10, and 10x, represent the type of various points and blades with serrated ends, all of which are bent to numerous angles and curves, and of different sizes, even to the most delicate.

Nos. 11 and 12 give the type of another variety of round instruments, ending in serrated points bent at different angles, and reduced to the most delicate dimensions.

Nº 13. Nº 14.

Nº 14X.

No. 13 is a type of which Nos. 14, 15, 16, 17, 18, 19, and 20, are modifications, for introducing gold into approximal cavities: the serrated point turning at right angles with the instrument, being made more or less prominent, according to the various presentations which may occur.

Nos. 14 and 14x represent a front and side view of one of the last named instruments, with a shallow plugging disk.

Nº 15. Nº 16.

Nos. 15 and 16 represent a right and left instrument. Being diverted to a slight angle, it becomes one of the most convenient instruments for introducing gold into approximal cavities, particularly between the front teeth.

Nº 17. Nº 18.

Nos. 17 and 18 are smaller instruments of the same character.

Nº 19. Nº 20.

Nos. 19 and 20 are especially useful in introducing gold into the anterior and posterior approximal cavities of bicuspid and molar teeth. These instruments are copied from patterns of Prof. C. A. Harris' suite of instruments.

Nº 21

No. 21 is an instrument whose extremity, or working end, is at right angles with the shaft, and terminates in a serrate form, much resembling a rake with a double row of teeth.

Nº 22. Nº 23.

Nos. 22 and 23 are modifications of the same instrument, diverted to opposite angles, making them rights and lefts. They are especially useful in filling front teeth.

Nº. 24. Nº 25.

No. 24 is a modification of No. 8.

No. 25 is a convex, dentated instrument for condensing the grinding surfaces of large stoppings, and is one of Dr. Hunter's patterns. By rotating the instrument when force is applied, each dentated point acts successively as a lever and a fulcrum, giving the instrument a power of condensation possessed by no other. The dentated points are represented too fine.

No 26.

No. 26 represents a cluster of points, some of which may be new to the profession. Those terminating in the hook form, *f*, *g*, *h*, *i*, will be found very useful in packing crystal gold about the joints, within the cavities of front teeth, when the operator is working toward himself. The wave form point (*j*, *k*,) is useful in filling all classes of front teeth. It is especially useful in the operation illustrated by Fig. B, hereinafter to be described. The uses of the other forms will suggest themselves to the eye of experience, and they may be modified or increased, to suit the needs of the operator. Several of them should be made rights and lefts. Most of them, with the exception of the point, may be advantageously constructed like Fig. 4, in the plate, which is made entirely of steel, and is one of the cheapest forms. All the modeling points, too, might be attached to similar shaped handles.

If the instruments are larger, the size of the steel handles may be proportionately increased. If a more expensive handle is desired, the form and material of Figs. 1, 2 and 3, may be employed. Fig. 1 in the plate represents a form for the handle of a larger class instrument, invented by Dr. C. W. Ballard, of New York, editor of the Dental Recorder. The engraving gives a very accurate idea of its proportion, and represents it of natural size. In certain cases it is very convenient and effective; being anatomically correct in its conformation, and adapting itself to the palm and figure of the hand so completely, as to ensure a remarkable degree of precision and certainty of movement.

For Nos. 10, 11, 12, 13, 14, 15, 16, 19, 20, and 25, this handle, made of ebony, is particularly useful. Fig. 2 represents a very convenient form for all that class of instruments, described as a modification of the modeling tool of the sculptor. It will be seen that it has the general character of that instrument of the artist. It is furnished with working points or presentations at either end, upon different curves, thus greatly facilitating the operation, and relieving the dentist of much of the annoyance and interruption consequent upon laying down and taking up the tools. Its points are serrated over their entire surface. For convenience, delicacy, and neatness of manipulation, for the readiness with which it takes hold on masses of crystal gold, the certainty and ease with which it carries them to their destination, and for all the uses for which it is designed, it has no superior. The cut represents the instrument with an ivory handle and of natural size. This, too, may be made cheaply by forming it entirely of steel. A variety of points are represented by the figure, (26.) All the modeling points already described may be attached to this form of instrument. Fig. 2 represents the instrument in profile; a front view of the same would represent the shaft of the instrument broad and flat, for a short distance below the point, to compensate for its thinness through its other diameter. Fig. 3 represents another convenient form of instrument for filling front teeth, well adapted to the points represented by Nos. 14, 17, 18, 21, 22, and 23. Its handle is made of ivory, with a silver ferule, the the same as Fig. 2. Fig. 4 is a cheap form of instrument, all steel, which, by increasing its size, may be adapted to all kinds. It would be well in any event to have two or three dozen of these instruments on hand. The point has been described and represented at No. 3. Three or four of this form of instrument would be desirable, the point of *this* being the largest—the smallest about one-fourth or one-sixth of its diameter.

If the dentist has any difficulty in procuring the instruments herein described, or wishes to study a still more rigid economy, he can easily make them himself—most, if not all, of which he may obtain by working over such as he may have on hand.

I have now described all the forms of instruments used by myself in the management of crystal gold.

It is necessary to be provided with several sizes of the various instruments. Although a description of many new instruments has been given, comparatively few of them will come into use in ordinary operations—oftentimes not more than two or three. The superior judgment and ingenuity of many who will have acquired experience in the use of crystal gold, may suggest better forms than many herein described, but the principle of their construction must be founded upon the character of the modeling tool of the sculptor.

### SPECIAL DIRECTIONS.

The method of opreating upon the various classes of carious teeth, as they present themselves to the dentist, will now be described, pursuing the order mentioned above. Suppose we are provided with a suite of the instruments described, with crystal gold and all necessary accessories for the operation. An ordinary cavity, in the grinding surface of a superior molar tooth, has been prepared for the reception of the gold. Nothing is better for drying out a cavity than a coil of soft tissue or French bibulous paper. To prepare it, soften by rubbing between the hands, then tear off strips and roll them into coil. Having with a sharp-bladed knife cut the crystal gold into blocks, strips, and pieces, to suit the character of the cavity to be filled, place the napkin around the tooth—some use bibulous paper for this purpose with great success. Then, with the coils of paper, thoroughly dry the cavity.

Now, with instruments 1, 2, 3 or 4, according to the nature of the presentation of the cavity, press slightly upon one of the blocks or pellets, which causes it to adhere; then carry it carefully to its place, fix it in its position, modeling and pressing it to all the inequalities of the cavity, by changing the presentations of the instruments used; then with various sized instruments, of the character of Nos. 10, 10x, 11 and 12, go over the entire surface to ensure its complete consolidation; or add another layer, before proceeding to condense with the smaller instruments, and thus continue to add layer upon layer, following between each with the smaller instruments, even with ultimate points and blades, according to the necessity of the case, until the cavity is full; by which means, a uniform consolidation is ensured throughout the entire substance of the stopping. Though not so plastic as clay, the operator will often be reminded of that material, in the use of crystal gold, from the ease with which fragments of it are taken up by the tools, and the readiness with which it is made to adhere to, and be resolved into, the texture of the gold that has preceded it. Now pass over the entire surface, with a condenser of the general character of No. 25, which, although it makes but little impression upon the already condensed gold, ensures an absolute uniformity of surface. Then trim down with a file, or burr-headed cutters, followed by Scotch stone, clear the surface with finely pulverized pumice stone; then follow with fine crocus upon a pine or cedar stick, and burnish. The result will be a stopping of absolute integrity, of unparalleled density, which will never change in the mouth, can never be removed, except by being cut or drilled out, and whose surface will remain bright and unbroken, more completely so than would pure cast gold, under the same circumstances.

Large cavities in the lower molar teeth, come next in order. These operations are generally surrounded by more difficulties, chiefly in consequence of the cavity of the mouth, retaining the flow of the saliva from the superior and inferior maxillary glands, and retaining it in such close proximity to the teeth, to be treated. During a lengthy operation, napkins will have to be repeatedly changed, and the saliva pump or some similar instrument, will come into constant requisition.

Suppose the case of a large molar cavity, nearly all the bony part of the crown gone, leaving only its walls, which are sound and healthy.

We shall use A. No. 2 gold for this case, it being a little more dense than No. 1, and we can pack it more rapidly. We cut it into blocks of various sizes and thicknesses, compress some of the larger of these, so that their original thickness is reduced one-half; these we shall use to make the first layer, to facilitate the operation, and ensure us against breaking through the thin plate of dentine between the bottom of the cavity and the living nerve beneath. With instrument No. 1, we take up one of the one-half condensed pellets, carry it to, and fix it in, its place. With Nos. 4 and 5, we model and condense it into all the inequalities; then follow it with such fine instruments of the shape of Nos. 9 and 10, as circumstances shall require, taking especial care to secure good *joints*—always being assured that the centre never suffers from over attention to the circumference—using as much pressure as the tooth will bear, to ensure the perfect integration of the gold. Another and another layer succeeds in the same order, until it is built up to the top of the cavity, taking care, whenever lateral pressure is required in the progress of the work, to give the lateral force *direct.* We fill the cavity flush, or over full, apply a condenser of the shape of No. 25, and finish as before described. If, on closing the mouth, any of the upper cusps strike upon the gold surface, we cut out the gold with a round graver, with burrs or drills, until the articulation is natural.

Suppose in the case just considered, that the tooth had lost not only the internal portion of its crown, but that the whole anterior approximal wall was gone, together with a portion of each of its frontal cusps. It is desirable to reproduce the entire loss, if possible. Most admirably does crystal gold adapt itself to this purpose. The extraordinary and remarkable facility with which it can be built upon itself, and formed into independent shapes, in any required direction, is no less wonderful than satisfactory.

In cases under consideration, where entire lost parts are being reproduced, it is necessary to extend the gold a fraction beyond the boundary intended to be occupied, to compensate for condensation and the necessary loss of substance in finishing. In the progress of building up, and modeling these independent shapes into form, pressure may be applied from time to time, upon the sides of the growing stopping, towards the centre of the tooth, with the same impunity that could be used upon the top. Especial pains should be taken to secure a good *joint* at its point of union on the sides of the tooth. Having introduced all the gold necessary for the operation, natural form is given it with files, gravers, &c., finishing as before with stones, crocus and burnisher.

In filling posterior approximal cavities in molar and bicuspid teeth, the usual preparations are made. Then, with instruments of the character of No. 1, we take up masses of gold on their convex or inner surfaces, of various thicknesses, adapted to the working spaces between the teeth, and carry them to their places, following with Nos. 19, 5, 8 and 10, of various sizes and angles; as each mass follows the other, we are both pleased and surprised to find how readily each new installment of gold takes hold of, and clings to, that which preceded it, the slightest contact often being sufficient to ensure this, thereby enabling us to withdraw the instrument, to model the gold in its place, or to get a new supply. After the cavity is a little over full, we pass over its surface again, and, looking well to its line of conjunction with the tooth, finish in the usual manner. Experience will soon enable us to make choice of the No. of gold best adapted to the various cases which may present themselves. The general rule is, to adapt the No. to the strength of the walls of the cavity; using the low Nos. for frail cavities, and the higher for the stronger; though some use No. 1 for all cases, while others use No. 4 exclusively.

As the space between the front teeth is usually contracted, by way of preparation for filling them, it is necessary to cut the gold into thin layers, nearly corresponding in diameter to the cavity to be filled, and subdivide these into smaller pieces, for working into lateral points of the cavity. Everything being in readiness, with instrument No. 1, 6 or 8, we take up one of the thin pellets of gold, and carefully direct it to its place between the teeth, and then towards and into the cavity to be filled, so far as it is possible to do so with the instrument in hand. As a usual thing, it is desirable to introduce another layer of gold in the same manner, before proceeding to condense with smaller instruments. This, however, depends upon the thickness, as well as the No. of the gold. We now use instruments Nos. 7 and 8, following with any of the smaller forms used for foil, best adapted to the case.

All of these, however, should be serrated until we come down to ultimate points. Various forms of these will be found in the cluster No. 26. In introducing these smaller instruments into the cavities, particular care should be taken to carry the points entirely over the whole line of their borders, especially consolidating them *there.*

Layer succeeds layer, each one being taken up by the other, and integrated into the whole, continual care being taken to calk well the joints, until it is filled flush with the sides. We now pass over the whole surface with a small size of No. 8 or 21, or with a pointed instrument, then with a strong and steady hand pass a blade burnisher several times over the surface of the gold, and let the patient rest. In resuming the operation, we trim down the gold with a thin file, follow this with tape, laden with pulverized arkansite, or the finest emery; pulverized pumice stone succeeds this in the same manner, to free the gold from all grit; then we finish with crocus, wash thoroughly, and the gold is ready for the burnisher, which we give a thin coating of castile soap; before using again, we cleanse the surface of the gold, and we have a stopping, which for solidity, impermeability, unity and beauty, is far beyond any result ever produced with any other material.

I will now consider the three classes of teeth beyond the reach of foil to save, referred to on page 256.

There is a two-fold object in describing the extreme classes of teeth under consideration.

The first, is to illustrate the severe tests which crystal gold has been successfully subjected to, and the second, is to prove that if crystal gold will meet these extreme exigencies, which have never been successfully met before, it is abundantly qualified to meet all other cases in which foil can be used. The illustrations are all, with one exception, (Fig. Q,) taken from cases treated in the mouth—all but two of them, (Figs. Q and I,) being in the mouth at this time where they have been from 9 to 18 months; each of which now presents the same appearance in all apparent particulars, that it did on the day of its completion.

Fig. I, page 274, is a portrait a little "larger than life," of a tooth now in my possession, filled in January, 1854—was in daily use, and remained in the mouth until the latter part of the succeeding November, when it was extracted. It is a tooth whose entire crown, with the exception of a small portion of one of the cusps, was reproduced in gold. It has the same external appearance now, which it had more than a year ago, and I have no doubt from analogies which will be herein presented, ts internal character has remained unchanged.

As before stated, it is now over two years since I adopted the crystal gold of A. J. Watts, in my practice. In an article published in the American Journal of Dental Science, of January, 1854, I referred to several *tests* to which I subjected the crystal gold. It is with satisfaction that I now refer to another test—the *test of time.* By this my expectations are more than realized, not only in my own, but also in the practice of many of my friends, do I find the largest and most complex stoppings continuing unchanged, even in the slightest degree, after a trial of from a few months to more than two years. The fillings referred to comprise all classes, many of which could never have been made of foil. In my own practice, as much as three-fourths of the crowns were often reproduced in gold; in several instances the entire crowns, both of bicuspids and molars; and in numerous instances, frail bicuspids of the character already described, swelled the list; and yet, although many were filled in the early part of my experience with the crystal gold, in almost every instance success has been complete. In every case, with two exceptions, the surfaces of the stoppings remain unchanged, retaining their polish and density as at first; nor have their grinding faces in any way altered their shape; they being neither depressed by mastication, dissolved, or broken up, nor has there been any perceptibly leakage or permeating of fluids of the mouth, into either the gold or joints between it and the tooth. It has been my custom for about a year and a half past, when some of the patients who belong to my "free list," have presented themselves to have a badly decayed tooth extracted, occasionally to relieve its present pain by treatment, and then to fill it with crystal gold, with the understanding that the tooth shall ultimately be mine. I have now something like 20 or 25 teeth out on *probation.* Of course, I do not expect to get but a part of them into my possession, but I have been so fortunate thus far as to obtain nine of them; including three filled with the intention of having them remain, but which I was obliged to extract; one in consequence of a severe blow upon the face of the patient, another from scurvy and necrosis, and the other in consequence of exostosis.

As might have been expected, these teeth were subjected to the most critical examination, and the severest tests. Passing a pointed plugging instrument over the entire surface of each, especially at the joints, all were found to be perfect, save two.

In each of these, the gold filling, for a small distance on its border, permitted the instrument to pass through its texture to near the bottom of the cavity, owing to the gold not having been completely consolidated at these points. Removing this porous part, the borders of the gold around it were found to be solid, and apparently impervious to the fluids of the mouth. The other case was similar to the one just described; the soft portions were filled with water and mucus, and were easily removed, while the far larger portion remaining was dense, entire, and in all respects fully answering the purposes of a stopping, showing most manifestly that a uniform care and thoroughness in filling the tooth, would have produced a uniform and complete result. There can be little doubt, that in each of these instances, the fillings would in time fail to preserve the teeth. I plead in extenuation that these were among my first efforts with crystal gold. My failure, however, no less than my subsequent success, proved what might, and ought to have been done.

Cutting one of the filled teeth in two, I removed the filling of gold. After heating it to redness, it was easily forged and rolled into plate, equal in all respects to any plate made of pure gold. To ascertain the structure and character of the gold within the cavity condensed against the dentine and walls of the tooth, a frail bicuspid, that had been worn in the mouth ten months, was selected. On chipping and cutting away nearly all of the surrounding walls of the tooth, leaving the stopping free and entire, it was gratifying to find that it was not only dense like the exposed surface, but that it presented an exact reverse impression of all the inequalities of the walls of the tooth against which it reposed. Taking a bicuspid, in which a large stopping had been worn for about a year, with a thin wheel of soft iron laden with emery, the substance of the tooth and gold were cut entirely through, making two transverse sections, thus showing the completeness of the interior character of the gold, as well as its admirable calking or stopping quality, as manifested at the borders of the stopping, where it united with the tooth. Nothing could be more perfect, the stopping being equally solid throughout its whole texture.

By the same process as above, a longitudinal section was made from grinding surface to apex, through a molar tooth, in which a larger crystal gold filling had been worn in the mouth for fourteen months. The stopping was, and is, as completely integrated and solid, from centre to circumference, as though cast from the crucible.

CLASS I.—*Frail teeth that have lost their anterior and posterior approximal surfaces, and have only their buccal and lingual walls remaining, (Figs. D. and F.)*

In these cases the lateral pressure of the instruments in introducing foil, and the expanded condition of it, after it is introduced, necessary to its retention and to secure a solid plug, are oftentimes sufficient to break the walls asunder; and even when they do not give way, they are, as has already been intimated, more likely than ever to be broken off by lateral forces, from the fact that the foil has a large leverage upon the frail walls, especially at their extremities, giving them a continual tendency to separate from each other. Fig. A represents a central, sectional view of one kind of this class. It will be perceived that the character of this tooth, admitted of its being excavated underneath the remaining portion of its grinding sur-

FIG. A.

face, so as to get a hold upon it, of a *dovetail* character. In filling the tooth, when the gold has accumulated to near these undercut places, it may be successfully introduced and packed within them, with the instruments of the character of Nos. 9, 10, and *b*, *e*, *g*, *j*, *k*, in Fig. 26.

As the operation advances, the gold becomes constantly and absolutely solid, so that by the time the cavity is nearly full, the opposing cusps are firmly strapped together with the *tie* of gold that stretches from one to the other. Further security is given to the whole by letting the gold accumulate flush over the surface, thus *inlaying* the deflected, crowning angles of the tooth; and also by packing the plastic gold over the irregular presentations of the external walls of the cavity, until it is flushed over its original outline, as represented by Figs. C, E and G. The stopping may then be filed out on its grinding surface, until it has a free articulation, and then be finished in the usual manner. If the walls of the tooth are unusually frail, the operation will be strongly fortified and ensured against accident, by inserting one or two gold screws with a short protruding head into the bottom of the cavity, as indicated by the white lines in Figs. A and B. As the crystal gold is introduced and condensed in the cavity, it lays hold of and becomes solid around them; so that their legitimate action and character is the same as though they passed through a piece of solid metal into the opposing tooth, and screwed firmly there, with the advantage of a perfect adaptation of the opposing substances to each other. Stoppings successfully treated in this manner become comparatively independent of the usual necessary requisites for their retention—since the little aid they receive from the frail opposing walls is far more than compensated for by the great protection and peculiar advantages derived from the character of the material used, and its wonderful adaptibility to their exceeding frailty.

The nerves of the class of teeth under consideration are generally dead; in which case they should of course be removed, and their canals filled with gold prior to commencing the operation upon the cavity proper. In the event the nerve is alive and healthy, smaller screws may be used, and inserted at an angle so diverted as to avoid interference with the more vital parts of the tooth.

FIG. B.

FIG. C.

Fig. B is a tooth of the same general character with the one just described; the opposing cusps are secured to each other by the cutting, or *dip* of the grinding surface. In some cases the layer of gold will necessarily be a thin one, especially if the tooth below articulates against it. Instances must be rare, however, in which the principle cannot be applied.

Fig. C gives a sectional view of a tooth whose opposing walls are bound together by cutting out, and taking advantage of a perpendicular undercut of its inner walls.

FIG. D.

FIG. E.

Fig. D is the highest type of that class of frail teeth mentioned on page 255. By natural and artificial means, an irregular grooving has been formed around the borders of the cavity, between the outer and inner walls of the tooth, so that when the plastic gold is solidified about them in the act of filling, it becomes insinuated and condensed into these depressions, thereby locking and banding the walls together.

Fig. E gives a transverse sectional view of the same case, illustrating the manner in which the grooving and irregularities serve to bind the cusps together. If the walls are unusually thin, a screw may be inserted, as represented by Figs. A and B.

FIG. F.

FIG. G.

Fig. F represents a superior molar tooth, of the same frail character as Fig. D. It is to be treated in the same way as the other, when, if skillfully secured, it has every prospect of remaining for many years, if not a lifetime, one of the most useful and cherished organs of the mouth.

Fig. G. shows a transverse section of the same tooth, illustrating the manner in which the gold lays hold on and secures the opposing walls of the tooth together. The concave groovings and projections on either side, are seen in the figure.

CLASS II.—*We now come to the consideration of a second class of extremely decayed teeth; namely, those which have none, or but a fraction of their crowns remaining, but whose roots are healthy, or are capable of being restored to health.*

Suppose we have a tooth to restore with gold, like the one represented by Fig. H. The entire crown is gone except its anterior approximal wall. Its roots are healthy, and, could a crown be permanently built upon it, in such a manner as to arrest the further progress of decay, it will become invaluable to its owner—perhaps as much so as though it never had been brought so near to sure destruction. Will it not be a triumph of art to save it!

FIG. H.

If the nerve is dead, let its canals be filled prior to attempting the major operation of supplying the crown, leaving the pulp cavity open, and enlarging its capacity at the bottom, to secure a lateral hold upon the base of the pillar of gold that shall spring up out of it, to the support of the crown which we are about to form. In the event it is desirable to attain still further security, it may be obtained by introducing screws, as represented in Figs. A, B and J; or, what is better still, by undercutting a broad shallow cavity, occupying three-fourths of the diameter of the tooth, as represented in Fig. H, which represents a tooth of the character under consideration, without the pulp cavity being exposed. The cavity is dry and protected. We are now ready for the operation. Gold is first packed and thoroughly consolidated into the pulp cavity, if exposed; if not, into and within the over-projections of the undercut presentations of the cavity. As the operation advances, a solid column of gold firmly established at its base, rises up out of the subcavity, over-lapping its borders, spreading out and covering the exposed dentine and projecting flush over its edge. We proceed, alternating with delicate and heavy instruments, and with rare and denser qualities of gold, (the highest numbers being mostly used,) as directed on page 266, gradually embracing, enveloping and inlaying the remaining portion of the crown, until we have built up the whole to a sufficient height to meet the end desired. It is then filed and carved into form, and finished in the usual manner. If the stopping should become wet during the operation, by faithfully following the directions in regard to misfortunes of this kind on page 253, we shall recover ourselves with the loss of only a little time, and the expenditure of a little more skill.

FIG. I.

Fig. I is a portrait, as before stated, of a tooth whose entire crown, with the exception of a small portion of one of its cusps, was reproduced in gold; its nerve cavity was not filled, but in all other respects it was treated in accordance with the description of the foregoing. After remaining in its place subject to mastication, and the severe test of an unusually filthy mouth, for a period of nine months, it was extracted—being one of my probatory cases—and was found to be as solid, unchanged, and perfect, as at the time it was filled.

Fig. J represents a sectional view of a bicuspid tooth with only its outer cusp remaining. The gold is secured to the roots by a screw, together with the hold that is gained by the insertion and consolidation of the material into the pulp cavity, which has been enlarged for the purpose, as described above. By a judicious disposition of the irregularities of the cusp to the purpose, it is secured to, inlocked, and permanently embraced by the gold.

FIG. J.

FIG. K.

Fig. K shows the manner in which a tooth that has lost its entire crown, is prepared for the reception or engraftment of a "*crown of gold.*" The pulp cavity has been enlarged at its base, so as to give it the broadest diameter at that point, as described above. For the purpose of giving it further security, and ability to resist the lateral forces of mastication, we have drilled four holes corresponding to the four angles of the tooth, about equidistant from the pulp cavity and the outline of the tooth; with a *tap* a screw is cut within each of these for the reception of a golden screw, which is so constructed that when inserted, and established in its place, its head, in the form of an inverted cone, shall prominently project above its surface, as represented in the figure.

The four screws are fixed to their places, all parts are thoroughly dry, and we proceed. The pulp cavity is now filled, and the accumulating gold flushes over its borders upon the upper surface of the dentine. We now pack gold Nos. 2 and 3 thoroughly about the screws, at the point of their insertion with the tooth, insinuating the material into their irregular surfaces, and making the gold of equal density with them. As the solidified material accumulates, it gradually spreads out, impinges upon, is enveloped and embraced by surrounding portions, when all are resolved into the unity of the whole. We keep the gold flush over the margin of the tooth, to compensate for lateral pressure and condensation during the operation. A good and uniform foundation having now been secured, gold of Nos. 3 and 4 is now placed upon it in broad thick masses, or blocks, each covering the entire exposed surface; these are successively and thoroughly condensed to their place, until the whole is of a proper and natural height, when it is filed and carved into form, and finished as above.

FIG. L.

Fig. L gives the general appearance of the tooth just described, after it is finished; but the crown is represented as being a little too high, and not as well formed as it ought to be.

All operations of the character described under this class, must necessarily be long and tedious ones. Sometimes they will be interrupted and deferred by getting wet. The *triumph* that lies in the end, should ever encourage us to press forward with an energy that *will not* fail. "Try, try again!" should be our motto, until we have secured the sure reward.

It took nearly *four hours* to perform the operation represented by Fig. I, and, from the fatigue of the patient, it necessarily got wet about the middle of the operation. After letting the patient rest, the gold was treated in the manner described on page 253. For the purpose of securing a mechanical hold, as well as that arising from the natural adhesion of the material, the condensed gold already in, or rather *on* the tooth, was drilled and cut into, with the same impunity that you could into any other form of solid gold. On resuming the operation, the first layer of gold obliterated the outline, or the *surface* of the gold first introduced, by the new installment being completely worked and integrated into it. I have no doubt, that were it removed from the tooth, annealed and rolled into plate, it would show no more inclination to crack, or separate at this point, than at any other.

The reader has no doubt ere this, been ready to inquire why this tooth, so successfully treated, was not suffered to remain, and if there was any necessity for its extraction. I answer, there was a *present necessity*, per pre-arrangement! I intended, as before stated, to have it ultimately come into my possession, and laid my plans accordingly. It will be recollected the nerve cavity was not filled, although exposed. After being treated for its "present infirmity," leaving the dead filaments of the nerve in its canals, a piece of gold plate was laid over the pulp cavity to answer for the bottom of the cavity proper, which was filled as above, with the expectation that the first severe cold taken thereafter by the patient, would result in arousing the latent energies of the semi-artificial organ to a hopeful tooth-ache. This did not transpire, however, until about nine months afterwards; even then, the tooth might have been restored, by passing a drill below the festoon of the gum and perforating into the pulp cavity, thereby giving vent to the accumulated gas there, and following up by further treatment. It was one of these sacrifices I felt justified in making for the advancement of our art. It is a representative of scores of others treated in the same general manner, now worn in the mouth of patients, most, if not all of which, I have no doubt, will remain during their lifetime. Such operations are triumphs, and I never look upon them, either in others practice or my own, without regarding them as results which while they are beyond all expectations hitherto entertained, approximate our profession, in this direction, quite to perfection itself; for we do in effect virtually reproduce and permanently *restore* to our patients a natural vitalized organ, capable of discharging all its normal duties, and of exercising all its important functions.

CLASS III.—*Bicuspids with Artificial Cusps and Crowns combined.*

In these cases, as with Figs. K and L, we have nothing but the root of the tooth to build upon. Although an entire crown of gold will answer all practical purposes, yet when located in the front part of the mouth, so as to be readily exposed to view, their appearance is far from agreeable. The application of an artificial cusp, resembling nature, to the external presentation of the gold, very naturally presents itself to the mind; and to accomplish this end, various plans have been successfully adopted, some of which will be described.

A root of a bicuspid tooth is prepared in the usual manner for the engraftment of an artificial crown—save the drilling out of the nerve cavity to serve as a pivot hole. A thin cuspidati plate tooth is selected and adapted to the buccal border of the concave groove of the fang. The artificial cusp is now covered with a thick lining of gold, as for plate-work. A piece of plate of the same thickness, and nearly the same length and breadth with the lining, is now bent from its centre lengthwise, so that the two deflected sides thus formed are nearly at right angles. To the bottom of and at right angles with these is soldered a round piece of plate, about one-half the diameter of the breadth of the lining; this is perforated with a hole for the reception of a screw. The convergent angle of this fixture is now placed, base downwards, and in the same plane with the bottom of the lining, against the backing of the tooth, perpendicular with its central line, and soldered there. A section of the fixture thus formed is represented by Fig. M. This is now placed upon the root to which it is to be attached; guiding it to the exact position it is to occupy, a drill is passed through the eye of the lining at the bottom, into the substance

FIG. M.

FIG N.

of the tooth a sufficient distance, to firmly hold the screw that shall take its place. Another hole of the same size is drilled into the root near its lingual border; a screw is cut within both of these, and a substantial golden screw fitted to each. Fig. N represents the relation of the fixture to the prepared root prior to the operation, though the screws are too small.

We are now prepared for the operation. All parts have been made thoroughly dry; gold Nos. 2, 3 and 4, A, cut into appropriate pellets, is before us. The lingual screw is firmly established in its place, when the gold is packed around its projecting head as directed on page 275, until it is enveloped by a broad cone of solid metal. One or two uniform thin layers of gold No. 2, A, covering the whole base of the root, are now partly consolidated to their place. The covered buccal drill-hole is now freely opened, when the prepared cusp is fixed to its position, and forcibly established there with the remaining golden screw, which, if thoroughly done, will render it sufficiently consolidated to justify us in proceeding. Gold is now freely carried to its place and condensed there, as described on page 275. As it accumulates, a portion of each successive stratum of gold lays hold on the diverging wings of the lining, and continually binding and enveloping it into its solid texture, it ultimately embraces and overcaps the whole. A column of gold rising up out of the nerve cavity, may often be appropriated in these cases, as also the undercut method illustrated by Fig. H, page 273. A better form of lining since adopted, is represented in section by Fig. Nx.

FIG. Nx.

Another method is to line a tooth in the manner represented by Fig. N without the diverging wing, and then solder a band of gold around it, so that it will correspond to the presenting outline of the root to be covered. Fig. O represents a sectional and Fig. P a perspective view of this fixture. The manner of establishing it in its place, is essentially the same as already described in connection with Fig. N. This method of operating combines great facility with neatness of execution,

FIG. O.

FIG. P.

and is generally adopted in preference to all other methods for this purpose. A gentleman of my acquaintance has, for several months past, worn an artificial cusp and crown of this general character, upon a root which contains undisturbed its *living* and healthy nerve. By accident the natural crown was broken off, but in such a manner as to leave a large portion of the central part of it covering the nerve, and which protruded down so far, that, by cutting a groove around its base, it somewhat resembled an inverted cone; gold was packed around this until it nearly reached the outline of the root, when the prepared cusp and crown, Fig. P, without its staple or screws, and which had been previously fitted, was secured to its place. The *tubbing*, or gold-bound cavity, was then filled with gold, as described above.

I doubt whether the annals of the profession can furnish an instance like this; certainly no material but crystal gold could have been the agent to accomplish such a result.

FIG. Q.

Fig. Q represents a form of an artificial cusp adapted to this operation, which could easily be made by the manufacturers of teeth. The cut sufficiently explains itself. Another method herein to be described, will, in most instances, take the place of either of the above for rescuing this class of teeth.

Although numerous teeth treated as above are successfully worn in the mouth at the present time, my object in referring to them is mostly for the purpose of illustrating the remarkable qualities and capacities of crystal gold, which enable it to successfully meet and overcome such extraordinary contingencies. To the most casual observer the inference is plain, that if crystal gold can be made to answer such unusual purposes, and successfully endure such unparalleled tests, it must unquestionably be a superior material for all ordinary purposes.

It is well known to the profession, that could we arrest, or be insured against, the decay and disease of the roots of front and some classes of bicuspid teeth, the method of inserting and engrafting artificial crowns upon them, would be adopted in every instance where it is possible to do so. When properly selected and skillfully adapted, they far exceed in naturalness of appearance, convenience and utility, any other method that has ever been adopted. The only practical objection to this method has been, the decaying of the root and consequent uncleanliness arising from it. I think I am correct in saying, that except in the practice of a few, pivot teeth are always regarded as at best of a temporary character, and that they are inserted in this manner with the confident expectation that at no distant day, they will be removed to be replaced with teeth on plate. This need not be; roots of this character may be permanently saved and made to answer for a lifetime, all of the essential purposes for which they were intended. A few have adopted for their preservation substantially the same treatment with foil, that has been more successfully practiced with crystal gold.

As the entire system of treatment referred to, will be fully embraced in a description of the manner of treating an extreme class of decayed roots about to be considered, it is deemed unnecessary to extend the notice of the above for the present.

When the crownless roots of front or bicuspid teeth are decayed, if treated at all, they are generally removed, no matter how healthy their external membranes, or firmly they are established in their place; if they have but the plague-spot of decay upon them, they may not share the remedy that would have been freely extended to their crowns, but must be summarily sacrificed to the expediency which knows no other remedy. But there *is* a remedy—an invaluable one; such roots can be fully restored, so as to retain the character, position and usefulness they once enjoyed, and even be made far superior in regard to permanence and cleanliness, to any other.

The cementum, being the connecting link between the low organized dentine and the periosteum, is highly vascular and vitalized, and so far resists the action of decay, that it never, while living, yields to its influence; when the supporting dentine has been decomposed and removed from its inner surface, its thin walls are broken down by mechanical influences, and in this way it is destroyed. We shall take advantage of this provision of nature, to ensure a peculiar permanency to our operation.

FIG. R. FIG. S. T. U. FIG. V. FIG. W.

Fig. R. represents a central, longitudinal section of a root of a frontal incisor; it is much decayed; the thin brittle edges of its deep cavity scarcely rise above the gum. The figure represents the cementum, the dentine and the nerve cavity. The decay has been removed, the nerve canal is now enlarged for a short distance, and a screw cut within it for the reception of a golden screw. The nerve cavity is now cleaned out and filled to its apex with gold in the usual manner, when it will resemble the appearance of Fig. S.

A cylinder of gold is now made for the reception of the pivot of the tooth, and a screw cut on the outside of it, with a thread corresponding to that cut in the root; when completed it will be of the form of Fig. T. If desirable, the upper part of it may terminate in a gold pivot of lesser diameter, placed for a short distance within the cylinder and soldered there, (Fig. U.)

All parts being dry, one of these cylinders is now screwed firmly to its place, so that its lower end shall be on a line with the border of the cavity, when gold Nos. 2 and 3, quality A, (some would prefer the Nos. quality B,) is now carried to the bottom of the cavity, where it lays hold upon its irregularities and the lateral presentations of the screw; alternating from large to small instruments, the gold is thoroughly consolidated to its place, care being taken while working about the thin edges of the cavity, not to break down the cementum. Gold A, No. 1, in consequence of its mellow, corky character, will be very safe and useful for sealing and calking the borders of the stopping near the conclusion of the operation. The cavity is filled a little over full, when the patient is allowed to rest. Fig. V will represent a sectional view of the operation at this stage. The gold surface is now freely filed, pared down and adapted to the crown which is to repose upon it. The gold cylinder enclosed by the equally solid gold around it, seems resolved into its texture, and the whole presents a non-corrosive and indestructible surface, as represented by Fig. W. It will readily be seen that no part of the tooth is exposed except the cementum, which, as before remarked, is so highly organized that it is not liable to decay.

There can be no doubt but that decayed roots successfully treated in the manner described above, are far superior to healthy roots treated as heretofore. They immediately become permanent and useful in their character, and so entirely are they protected from all external influences, that, unless some severe accident befalls them, their chances of life are about equal to that of the patient himself. Several have adopted this method of treatment for all classes of pivot teeth. Ultimately this plan must, I think, be universally approved. This is the "other method" of preserving the roots of bicuspid teeth for the reception of artificial crowns, referred to on page 279. In such cases, an artificial cavity is cut out of the base of the root from its centre to the cementum at its circumference, when it is treated as described above. It is desirable in all instances, where it is practicable, to make *undercut* excavations to aid in retaining the gold, though when screws are used, this is not indispensably necessary. A few adopt this course of treatment with all roots intended for the engraftment of crowns, whether healthy or decayed.

Some form a broad, slightly undercut cavity, fill the whole with solid gold, (A or B,) Nos. 3 or 4, and then boldly drill into and through the centre of the gold to form a pivot-hole. If the operation of packing is successfully performed, the stopping will endure the test as well as though it was solid gold of any other form.

Crowns may be secured to the roots of teeth as prepared above, either by wood or gold pivots, as usually practiced ; gold pivots are generally preferable. Some, however, prefer another method, as follows. After the root is filled with gold as above described, and properly finished, an impression is taken of its surface in wax, from which castings are made, and from these plates are swaged; these are adjusted to the roots, and a golden pivot is soldered to each of their upper surfaces. A plate tooth is now skillfully adapted to the fixture, when it is ready for use. In this manner a plate may be extended across an intervening space unoccupied by roots, and an unbroken row of teeth mounted upon it. It may be urged against this method of inserting teeth, that it must of necessity cost the patient three or four times as much as it would by the old process. It is sufficient to say in reply, that the method under consideration is tenfold more valuable.

It is more meritorious to restore one natural organ to health and usefulness, than to construct the most complicated work of art as a substitute for it.

Crystal gold commends itself to all classes of *shallow* cavities, especially if they have much breadth of surface. It is well known that in order to ensure the retention of foil in cavities of this character, it is necessary there should be a certain relation of depth to their breadth, to balance the lateral forces or bearing of the material against itself; else it will be continually thrown out in the process of packing and wedging. Crystal gold does not depend upon these lateral forces for the undisturbed position of its particles during the operation, nor for its retention afterwards; every succeeding layer becomes successively independent in its character, and continually aggregates to the support of the whole.

In consequence of these peculiar characteristics, broad cavities of extreme shallowness may be filled with every prospect of permanency and usefulness; for if the operation is skillfully performed, it results in supplying the entire lost portion of the tooth, with an impermeable material, as hard and durable as plate itself. I recollect having seen, several months ago, in the practice of Dr. Ballard, of New York, an interesting exemplification of this peculiar ability of gold. A superior sapientia of one of his patients, had commenced decaying before it was developed, so that by the time it was advanced to its position in the maxillary, the inferior molar beneath actually articulated so far within the cavity, as to press upon the thin layer of caries and bone that covered the nerve. The caries, covering nearly the diameter of the tooth, was carefully removed, when the whole was skillfully stopped with crystal gold. I have no doubt the stopping is as permanent in its character as any operation in our art; and yet, no one with a true appreciation of the case, could, for a moment, have deemed it possible to succeed with foil. I have often succeeded in preserving valuable roots of teeth, by forming a cavity within them, in which I could *anchor* a quantity of gold to serve as a nucleus, or foundation for the superstructure, and building over the entire cusps, a comparative thin layer, or *shoeing* of gold, which, thus far, effectually arrests the progress of decay, and renders them highly useful in their character.

Another illustration of the remarkable adaptation of crystal gold to peculiar exigencies, and we have done with this department of the subject. It has been repeatedly remarked, that while crystal gold is capable of being built up into independent shapes, it becomes absolutely compact in its character, whatever direction it may take, or position it may occupy. Its remarkable faculty of taking an exact impression of whatever it may come in contact with, has also been referred to. About six months ago, one of my patients desired me to secure the remaining portions of a valuable superior second molar tooth against decay. It had lost all of the posterior portions of its crown, nothing being left of it but a part of its anterior cusps, which, springing obliquely from the posterior gum-line of the crown on one side, and perpendicularly from its anterior side on the other, formed an acute angle at its articulating surface.

The remaining portions were solid and entire, save an *internal* cavity which extended from the main one, underneath and through to another, covering its anterior approximating surface, which reposed firmly against a neighboring molar. To pass a conical file between the teeth, to enlarge the space, sufficient to introduce gold from this direction, would result in removing too much of the already limited portions of the tooth remaining.

After passing a thin file between the teeth, I wedged dry cotton into the space thus made, protected the main cavity with wax, and discharged the patient until the next day. In resuming the operation, delicate excavators were used between them where it was possible to do so, but a major part of the excavation was made by instruments passed through the cavity from the back side. All things being in readiness, and every part made thoroughly dry, a broad thin piece of gold—made very dense by rolling—was wedged between the teeth, so that one of its surfaces braced firmly against the open cavity of the tooth to be filled. In the process of filling, when the gold had accumulated so as to be on a level with the approximating cavity, it was introduced from behind and through the internal cavity, and consolidated against the plate facing the anterior opening, special care being taken at this stage of the operation to give the material lateral directions with the instruments, throughout its entire circumference. The accumulating gold gradually approaching that in the main cavity, was soon absorbed by it, when the whole was completed in the usual manner, restoring to the tooth its original form and usefulness. When the plate was removed from between the teeth, I was gratified to find the anterior surface of the stopping hard, polished, and effectually insinuated into every crevice or irregularity of the margin of the cavity; the smallest pointed instrument made but little impression upon it, and in finishing it down, its surface, which was actually the *bottom* of the cavity, proved in all respects to be as dense as any part of the grinding surface of the stopping.

All fragments of crystal gold, whether wet or dry, can be appropriated to a practical use, so that none need be lost. Gather all such particles together in a mass, place them upon a thin strip of platina plate, and heat the whole to redness over an alcohol lamp; while in this condition, lay the platina upon some hard substance, and with a smooth-faced hammer press down upon the annealed mass, when it will be found to be quite dense, and comparatively tenacious. The material thus formed will be found to be well adapted for the first layer or *flooring*, for large cavities.

Owing to a peculiar quality of crystal gold, alluded to before, each portion of gold directly under the instrument, alone receives its pressure; in this manner, the gold is carried *through* the uncondensed mass, and consolidated against the wall of the cavity, or the already condensed gold. The pressure is not distributed throughout the substance of the imperfectly packed material as with foil. While these individual portions of gold are left free to the legitimate action of the point of the instruments, they are not only integrated into the texture of the whole, but at the same time each particle is successively *tempered* or *hardened*, as the operation advances.

By these means crystal gold may be made to acquire a specific gravity equal to platina itself. In order to ascertain what *could* be accomplished in this direction, I made a steel ingot, and drilled a hole into it ¼ inch in depth, and the same in diameter. Into this I packed nearly three dwts. of crystal gold. The operation was a long one, for I was not satisfied with simply integrating the gold together, but wished to make it as dense as possible. On testing its specific gravity, I found it to be 20.5; pure cast gold being 19.25, platinum 20.00, the latter when rolled and hammered, 21.5; so that my specimen is very near the gravity of platinum in its most condensed state.

I have thus far omitted to mention two tests that crystal gold has been subjected to by others as well as myself. Teeth that have been filled in the mouth, on being extracted, have been subjected to the action of muriatic acid, until they were entirely decomposed, leaving the gold stopping intact and unchanged. On other occasions, stoppings removed from teeth (made within, as well as without the mouth,) have been retained in various acids for months together, without in the least changing their character or texture.

To recapitulate, the causes of failure, may be attributed: *First*, to the use of an imperfect article of gold. *Second*, to the want of a true appreciation of the characteristics of the material. *Third*, to not having proper tools, or if provided with them, not correctly manipulating with them. *Fourth*, want of experience, and patience to learn. *Fifth*, to too rapid and careless operating. No one can succeed in the use of crystal gold, who attempts to fill fifteen or twenty cavities per day with it, as some do. The wonder would be, that such could succeed at all. Indeed, it is unreasonable to expect that at first, dentists can use crystal gold with the same facility that they can foil; and even were they never able to work as rapidly with it as with foil, it should be no cause of discouragement, so long as they can point to such superior *results*. This is the ultimatum for which we all are striving, the grand approximation to the highest perfection. Superior operations are soon known and appreciated, and the skill which produced them cheerfully rewarded. The fact that it requires more material to fill a given cavity with crystal gold than with foil, is but another evidence of the denseness and superiority of the stopping. But time, patience and practice, as in all other labors, here give us the sure reward of increased skill and facility, so that after a while, we are enabled, in most instances, to work with greater rapidity than with foil, *and always to produce a superior result.*

There are some in our profession who, from want of a thorough skillful experience in its use, have got an impression that stoppings made of crystal gold, however perfect they may have been at the time of their completion, necessarily undergo a change which in a few months results in their disruption and consequent destruction. They have seen indications which seem to justify them in the belief that all stoppings made of crystal gold would inevitably "disintegrate," "soften," "absorb the fluids of the mouth," "break up," "dissolve away and become like brickdust:" while at other times they have been alarmed at the excessive density of such stoppings, which, from their estimate of the laws of the expansion and contraction of metals, would of necessity result in the destruction of both tooth and stopping. Taking this highly philosophical view of the matter, they have very honestly warned the profession against putting this new "enemy in their mouth to steal away their" *teeth!* So firmly has this idea taken hold on the minds of some, that we are cautioned against making foil stoppings *too hard*, but so to temper their softness, that the "porosity of the gold will about equal the porosity of the dentine," very naturally suggesting to the mind of philanthropic inquiry the application of the principle of the *gridiron pendulum*, or some other system of compensation, for this new and extraordinary capacity of gold, which had hitherto escaped the attention of the whole scientific world.

Our gratitude to the distinguished discoverer is further enhanced by the ingenious and philosophical remedy which he proposes. The suggestion of comparative porosity, &c. &c., is somewhat vague—at least it involves delicate experiments to ascertain the absolute specific gravities of the two materials, and then to skillfully balance the difference between them. We are kindly relieved at this point by the most definite and lucid instructions. "Make your *stopping sufficiently soft as to admit of its being permeated by the fluids of the mouth, and you are safe!*" This is the golden mean between the two contending and dangerous influences. Be sure and get your stoppings *soft* enough, and your teeth will never burst open by the expansive force of the gold, nor will a gaping trench surround it by contraction. Never allow a stopping to leave your hands unless you are sure dampness can freely permeate it! What is more easy? The simplicity of the remedy is only exceeded by the object of our gratitude!

I have no disposition to rob the well earned honors from, or even to divide them with, the distinguished discoverer, but would humbly beg leave to suggest, that in case the operator should, through carelessness, waste of time, and over thorough manipulation, allow himself to make a stopping too hard, he might save himself the *labor* of replacing it with a softer one, by piercing it through to the bottom of the cavity with a few score of small drill holes. Dampness of the mouth could then freely permeate it, and when subjected to the *wide* range of the changing temperature of the mouth, the texture of the stopping would have a free play to expand and contract upon itself!

We are gravely asked "if gold is decomposed by the fluids of the mouth." Some have gone so far as to say that it is! We must coin new terms for our language, and henceforth chemists must speak of *salivates* and *hydrates* of gold!

A little reflection, however, with a due consideration of the character of the material used, and the laws which govern it, will be sufficient to banish all of these newly formed fears, and show that the cause for failure or want of success with crystal gold, lies altogether in another direction.

First of all, pure gold is pre-eminent among the metals for its immutability. There is no one primitive fluid or substance that will dissolve or separate its particles—nothing in which it is soluble. The chemical action alone, resulting from the latent forces arising from the combination of two of the most powerful acids, forming nitro-muriatic, are sufficient to resolve the integrity of its affinities, and suspend it in solution. Intense heat will melt it; under some extraordinary circumstances, it may be oxydized, and under others, as has been intimated, it may be resolved into peculiar conditions, but these never compromise its character, and did they do so, they could have no bearing upon the practical uses to which gold is subjected.

Its specific gravity is next to platinum and among the superior metals, ranks next to it in being least affected by the expanding and contracting influences of heat and cold.

To again recapitulate. Pure crystal gold will not change its absolute condition in the mouth. As it is left, so will it remain. If partially condensed and integrated, so will it remain, until its spongy, broken and half united crystals admit of its absorbing the fluids of the mouth, when, in the course of time, by the friction of mastication and displacement from softening of the impinging walls of the tooth, by the acrid secretions which it contains, it becomes disrupted, broken up and wasted. Originally it was a dry porous stopping; it immediately became a wet porous one; then the external influences of mastication, the internal forces of the changing fluids which it contained, operating mechanically upon itself and chemically on the surrounding bone, resulted in its being thrown out precisely in the same porous and partly consolidated condition in which it was placed there by the disappointed operator, who now wonders the spongy mass did not *get solid of itself!* The gold has undergone no absolute change; as it left his hands, so it is. He is now prepared to talk to you learnedly about its resemblance to "brick-dust," and complains that it absorbs the fluids of the mouth, breaks up and dissolves away.

Another takes the same material, and with time, patience and thorough manipulation, effectually integrates the constantly accumulating gold into the integrity of the whole, making it absolutely solid, as hard as coin, and impervious to any and all fluids that it may come in contact with. Such stoppings are capable, whether in or out of the mouth, of successfully enduring all the severe tests that have been referred to in this paper.

We are told that the constant expansion and contraction of the plug and the tooth, will cause any plug to give way, in consequence, we are left to infer, of the fluids alternately permeating about the margins of the cavity. Let us take this "bull (an Irish one) by the horns," and courageously look the monster in the eye; perhaps we may see there more of the same sound—*soft* philosophy which at first was so alarming to us. Pure gold, when heated from 32° to 212°, viz. from freezing to boiling point, a range of 180°, expands about $\frac{1}{700}$—one seven-hundredth—of its diameter. A range of 20° is a large estimate for any changes in the mouth that can be considered of a lasting character. The extremes of heat or cold are so quickly compensated for in the mouth, that only a fraction of this range of temperature, could ever have legitimate influence on the entire substance of a gold stopping located there.

Admitting, however, the above hypothesis, in a range of 20°, its expansion would be only between $\frac{1}{6000}$ and $\frac{1}{7000}$—one six and one seven-thousandth of its diameter. A gold stopping one inch in length would expand or contract under these influences $\frac{1}{7000}$ of its length, that is $\frac{1}{7000}$ of an inch. A stopping $\frac{1}{4}$ of an inch in diameter is a very large one, $\frac{1}{7000}$ of this is $\frac{1}{28000}$ of an inch, which, admitting the tooth to remain stationary under these same influences, would be the fraction to represent the space between the tooth and gold, a space so minute that no fluid less subtle than electricity could pass through it. I have turned from this for a moment to look through the microscope at the dental tubuli, and find by the micrometer, that on an average, they measure $\frac{1}{6000}$ of an inch in diameter, nearly five times greater than the space would be according to the above estimate. This is attenuated philosophy indeed!

But even these estimates are based upon the supposition that the tooth, though subjected to the same causes with the gold, does not share in its effects—that it remains unchanged, unexpanded, uncontracted, or at least that they are inharmonious in their *movements*. But who knows this? Where is there any data from which we can form an estimate?—Who knows but that they are affected precisely alike? The probability is, that they are very nearly so; in any event the difference between them could only be calculated by a fraction of millions. The natural elasticity of gold, no matter how dense, and more especially that of the teeth, would be sufficient to compensate for all conceivable or practical differences. But we weary of this unprofitable transcendentalism.

A few words to the profession, and this lengthy paper will be concluded. I am aware that in this treatise I have in many respects gone counter to the expressed opinions of several in our profession, whose experience with crystal gold has not been a fortunate one. However much I would like to agree with them, truth and my own convictions will not permit me to do so. Belief is not a matter of volition with us. We believe or disbelieve according to facts presented to the mind and to our senses. Our choice or our prejudices have nothing to do with it. It would be difficult to convince the many hundreds who use it generally, and the many scores who use it exclusively, that they are not successful with it. They have tried and tested it in various ways, and even though they would, they could not gainsay their own convictions.

It seems to me the height of folly for one man to undertake to make himself the standard of all human ability, and to urge his own *negation* as evidence against a living fact; to endeavor to do away, or to deny the existence of a positive or tangible truth by negative testimony; it is saying, I do not succeed, *therefore* you do not; others do not succeed, consequently no one does. This kind of reasoning in no way affects anything but itself. It is simply a *confession of inability*, and there it stops.

It is an array of what *has not been done* against what *has been done*. One perfect crystal gold stopping, by *whomsoever* constructed, establishes what has been done, and what can be done again.

A skillful operator reproduces a part or the whole of the crown of a tooth. It successfully endures all the varying influences of the mouth for years; it becomes a fact, a beautiful, palpable and substantial creation; there it stands, and everybody can see and know of it. How much negative evidence will it take to annihilate this fact—to crush it out of existence? It cannot be done: and this is one of the beauties of truth; no matter how humble the source from which it springs, the combined testimony of all the learned against it, can in no way disturb or rob it of one ray of its brightness. All the error this side of Pandemonium can never militate against a single established fact. Unlimited negatives do in no way affect the integrity of a single truth or creation; for if this were the case, every beautiful creation, every established fact, and every sublime truth, would be in continual danger of being torn down from its high position and destroyed.

If I know my own feelings in a professional point of view, I think I give truthful utterance to them, when I say, that I most and only desire the highest perfection in our art, and all the inventions, discoveries or auxiliaries in any form, that shall give a healthful impulse to our progress, or elevate our standard to a higher position, I hail with pride and satisfaction; and while I would commend to myself due caution before embracing anything new, I trust I shall be equally emulous of that spirit of fairness, without which no one is open to conviction. I recognize crystal gold as a very remarkable and most valuable aid to our profession, and as such I endorse it. What others say against it, should not, and does not, in any way affect my convictions, based as they are upon my own experience, and corroborated by the best operators in our profession.

I cannot conclude without saying a few words to the younger members of our profession, and indeed to all of those who have been discouraged in the use of, or have been dissuaded from, attempting to use crystal gold by the representations of those would-be oracles, who only exemplify that they do not know of what they speak; their complaints and warnings are at most but a confession that they are unable to do what others are continually and successfully doing. Be your own oracle; remember that what has been done can be accomplished again, and by *you* as well as by any one, if you but persevere. Do not estimate yourselves too lightly, nor pay too much deference to genius, that "mysterious gift," the contemplation of which so often discourages the hand and heart of honest toil. Be assured that the great difference in men after all, arises mostly from their different degrees of energy and industry, characteristics which all may acquire. Every successful production of yours is a silent and complete refutation of all that any one may say against it. Press forward in the glorious enterprise of perfecting our art, giving no heed to those who stand on the corners of the streets crying, "Woe! woe!" nor listen to the voice of that conservatism which would persuade you that the goal of human progress has been reached, and that further aspirations after knowledge are vain.

From among the numerous letters commendatory of crystal gold, which I have been permitted to peruse, I take the liberty of making the subjoined extracts:

Prof. Arthur, of Philadelphia, after speaking of the active opposition on the part of some, says:—"Instead of losing confidence in this material, (crystal gold,) every day's experience confirms more and more the opinion that I have expressed, that it is a beautiful and exceedingly useful improvement in the appliances of our profession."

Dr. Ballard, of New York, who, among others, used crystal gold exclusively in his practice, writes in one of his letters:—"I cannot let the sun go down without thanking you again and again for bringing an article we have so long desired to such a degree of perfection. If I can always rely upon getting crystal gold like your last, I shall never use anything else for filling teeth. The profession are under inestimable obligations to you for your invaluable discovery."

Dr. Hunter, of Cincinnati, writes:—"I consider the last lot of gold sent, the very *ultimatum* of our wishes. I do not see as you have left our profession anything to desire in an article for filling teeth."

Various attempts have been made from time to time to supply the profession with a form of gold which would be superior to, and take the place of, gold foil, but up to the time that Dr. Watts obtained his patent for the making of prepared gold, and thus made the results of his experiments known to the public, nothing of practical value had been produced.

During a long series of experiments, embracing a period of nearly three years, Dr. Watts, having discovered the principle or law of crystallography, which governs the organic forms of gold, has practically covered the whole ground of this new field of scientific research, and has found himself capable of producing almost innumerable varieties either in crystalline or aborescent forms. At least one hundred distinctly different practical varieties are producible in the field covered by his patent, and that whole field had been previously unknown to science. Every conceivable quality of texture, whether hard, soft, fine, coarse, harsh, tough, adhesive, brittle, elastic, silken or velvet like, he can produce at will, or give any gradation of texture by combining the above qualities in any desirable proportion. Not being himself a dentist, and not knowing which of the many varieties were most practical in the hands of the operator, Dr. Watts looked to the profession for direction. Here he found preferences to gratify, almost as diverse as his varieties, arising from the character of the tools used, and the system of manipulation of the operator; which greatly embarrassed his progress. Taking into consideration, however, the aggregate of choice, he settled down upon the manufacture of four varieties, distinguished by numbers, indicative mainly of their differing density.

The history of the discovery, progress and development of crystal gold under the hands of Dr. Watts, has illustrated that in the use of this preparation, as in other things, "practice makes perfect"—and that although, with care and patience, a very desirable end may be attained with it in the early part of a dentist's experience, yet the highest and most perfect results are only gained by the largest experience and the greatest degree of skill—the advancement of the one always keeping pace with the other. The more experience the operator has, the more skillful will he become in its use; the more remarkable will be his productions; the more knowledge will he obtain of the peculiar characteristics of the material; the more attached to it will he become, and the more indispensable will it be to his practice. Recognizing the necessity of this *probatory* system of education, Dr. Watts, for the present, retains from the profession, a more highly perfected article of gold, which he believes will ultimately take the place of all other varieties of crystal gold now in use. It will require a higher degree of skill, and a greater amount of experience for its successful use, than the profession have yet acquired. When, with the present forms of gold in their hands, they have educated themselves up to a point which will justify him in bringing it before the profession, he will do so—but until that time, if I may judge from my unsuccessful, yet urgent pleadings with him, he will not. He says in justification of his course, that it possesses such peculiar characteristics, that while in the hands of skillful experience, it will, with surprising facility, produce results hitherto deemed beyond the reach of art, yet in the hands of the inexperienced, it would very likely be condemned.

He now proposes to offer to the profession, eight different varieties or forms of his gold; four varieties under the name of "A. J. Watts' Patent Prepared Gold," and four varieties under the name of "A. J. Watts' *Improved* Prepared Gold." The numbers in each case, running from one to four, and indicating the varying density of the material, No. 1 as heretofore, indicating the most porous, and No. 4, the most dense.

All of Dr. Watts' varieties will be recognized under the general head of Crystal, or Crystalline Gold. The *Improved* Prepared Gold, will be styled quality A. The Patent Prepared Gold, quality B.

Many of the most successful operators with foil, succeed best when they prepare their gold in the form of pellets, which are constructed by breaking up the smooth surface of the foil into as many angles as possible, and shaping it into balls or pellets of different sizes; these are introduced one by one into the cavity of the tooth, where they readily take hold upon those which have preceded them, when they are gradually worked into their texture. Crystal gold comes to us in a condition vastly superior to any prepared foil in this respect, and in a form from which we can readily cut off pellets of any desirable size or character.

The forms of instruments numbered 15, 16, 17, 18, 22 and 23, and Fig. 3 in the plate are copied from Dr. Ballard's suite, that of No. 12 from Prof. Arthur.

*Erratum.*—Page 287, 12th line from bottom, for "disintegrade" read *disintegrate.*

FIG.1.

FIG.2.

FIG.3.

FIG.4.

## Figures and Tables

**No 1 f1:**
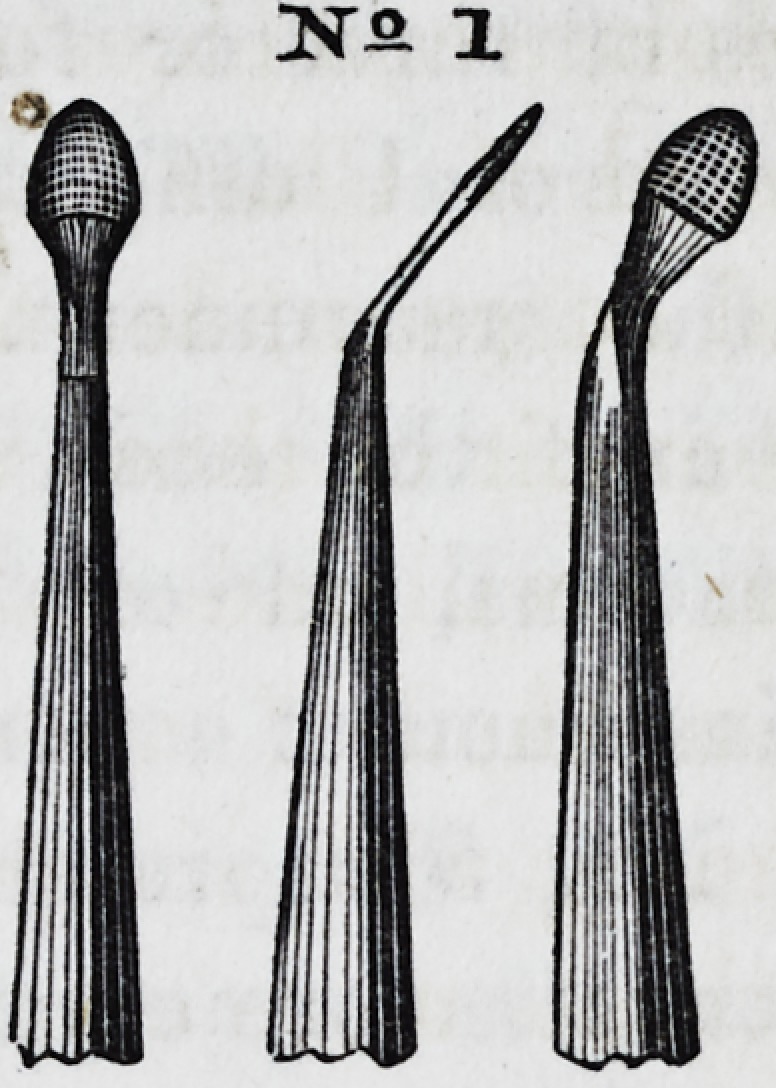


**No 2. f2:**
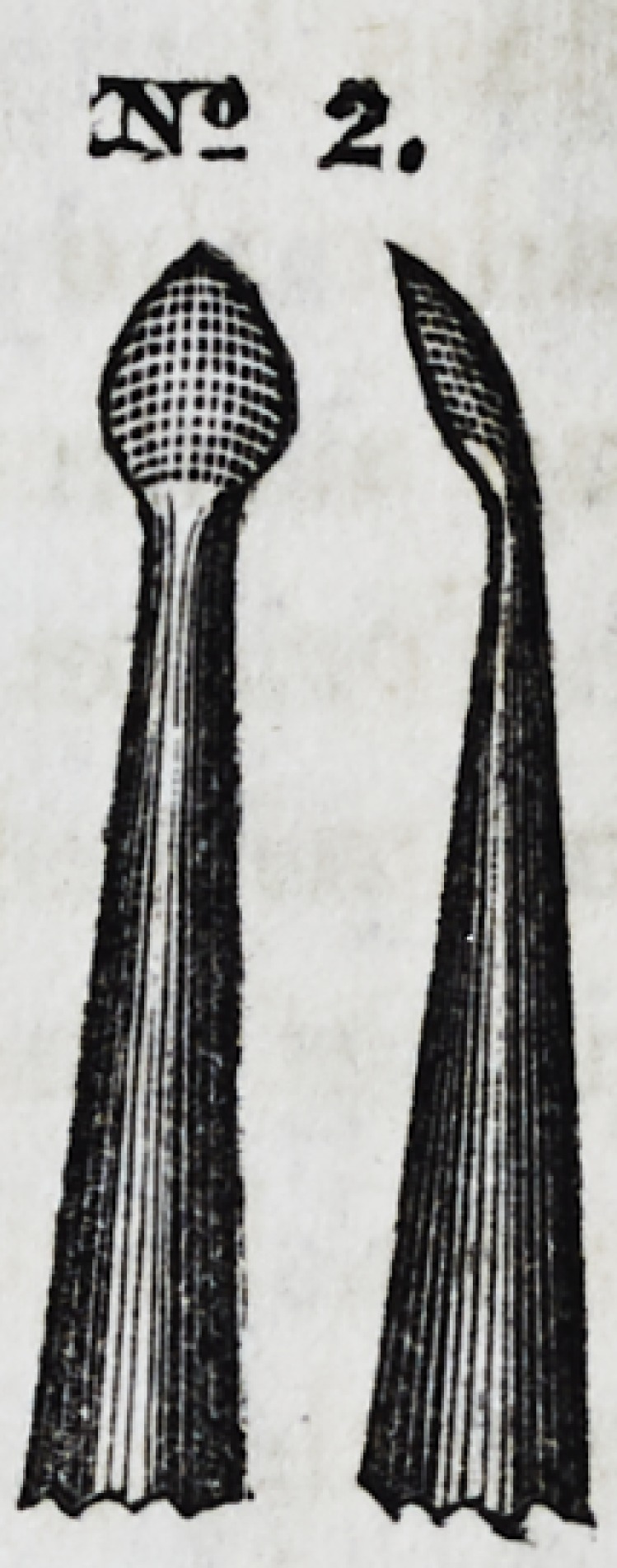


**No 3. f3:**
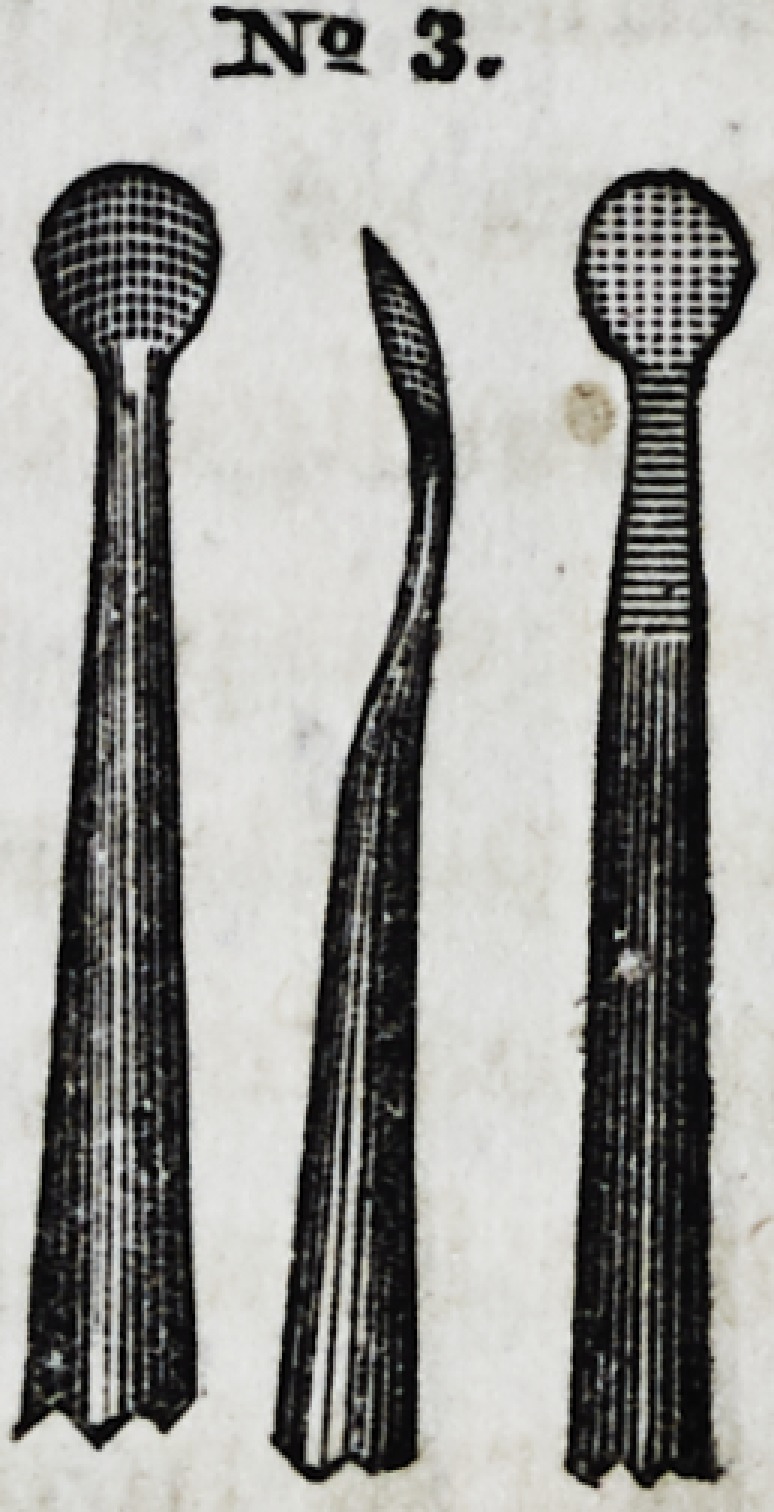


**No 4. f4:**
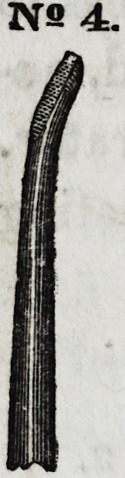


**No 5. f5:**
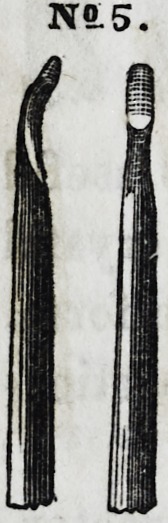


**No 6. f6:**
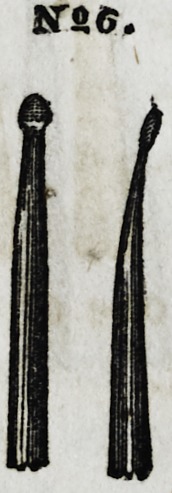


**No 7. f7:**
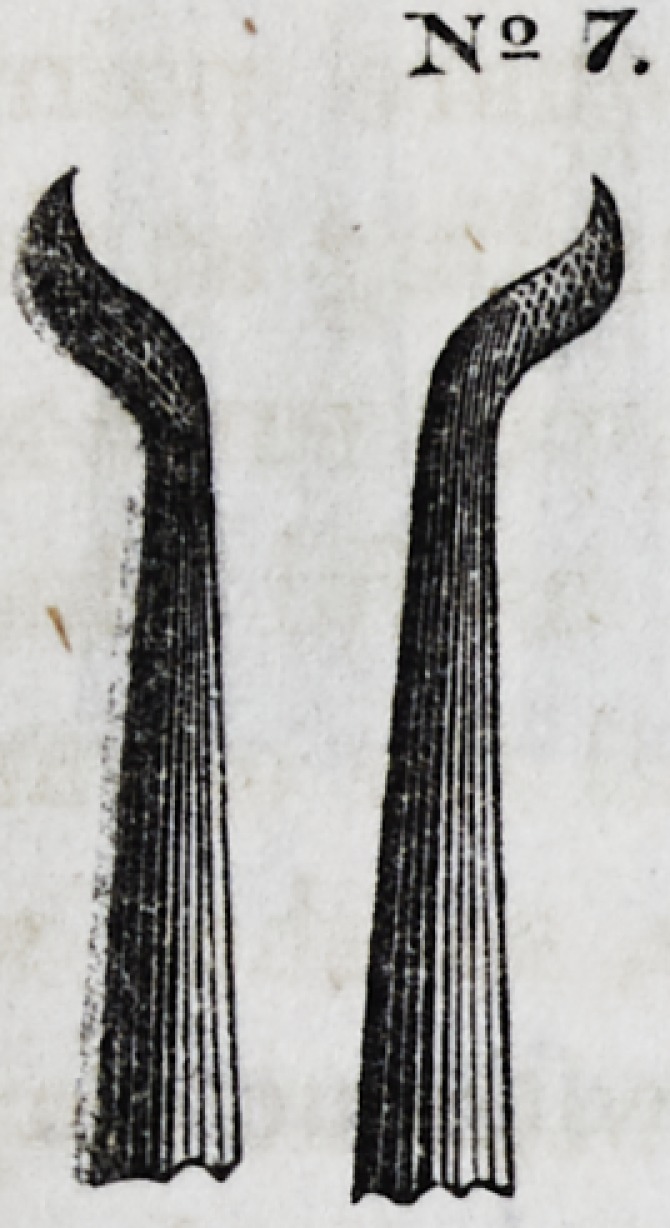


**No 8. f8:**
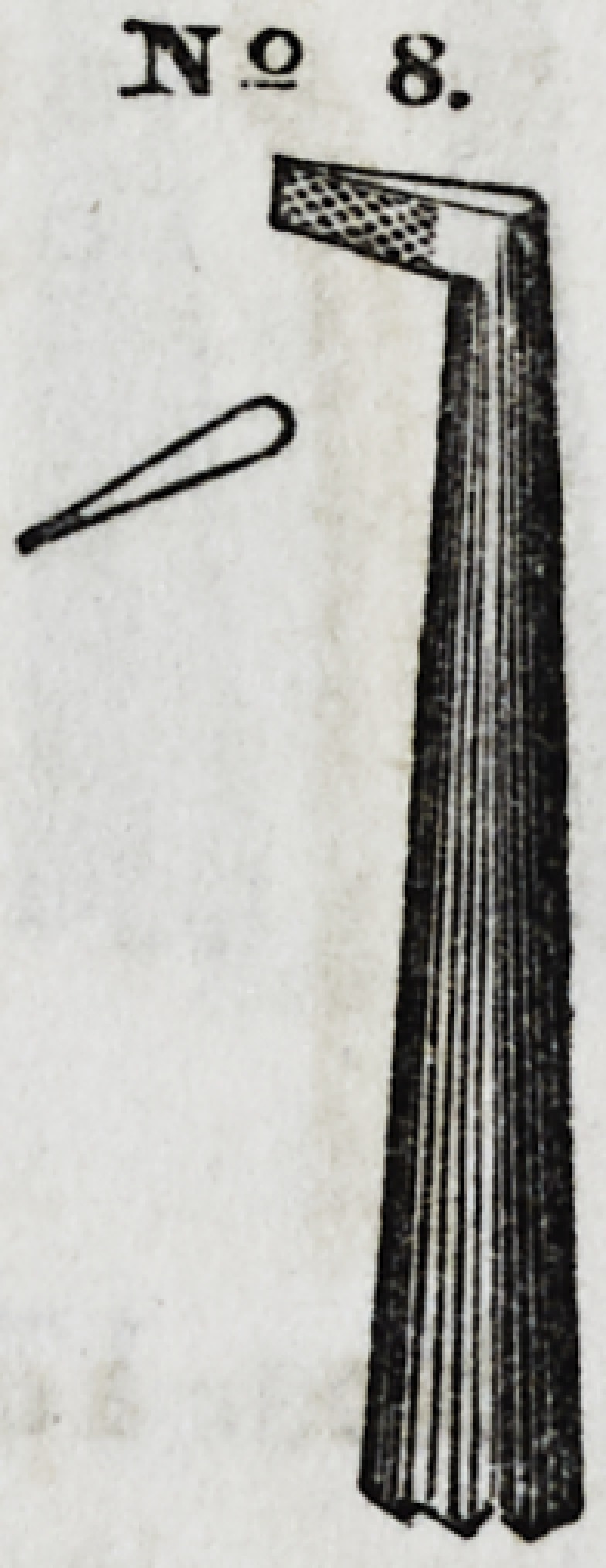


**No 9. f9:**
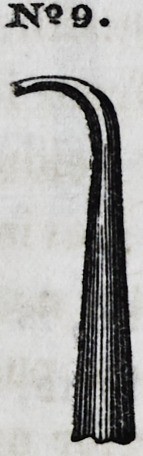


**No 10. f10:**
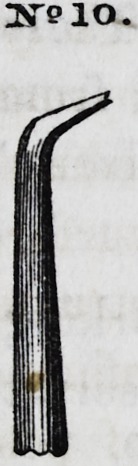


**No 10x. f11:**
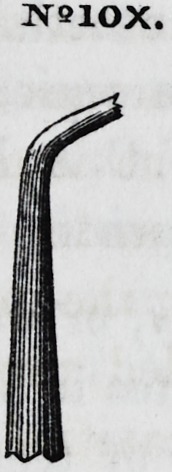


**No. 11. f12:**
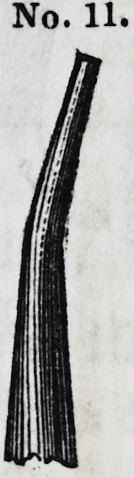


**No. 12. f13:**
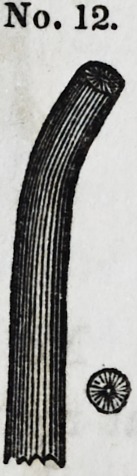


**No13. f14:**
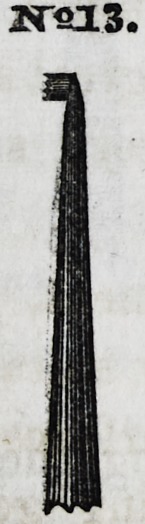


**No14 f15:**
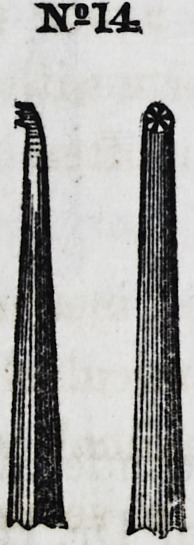


**No 14X. f16:**
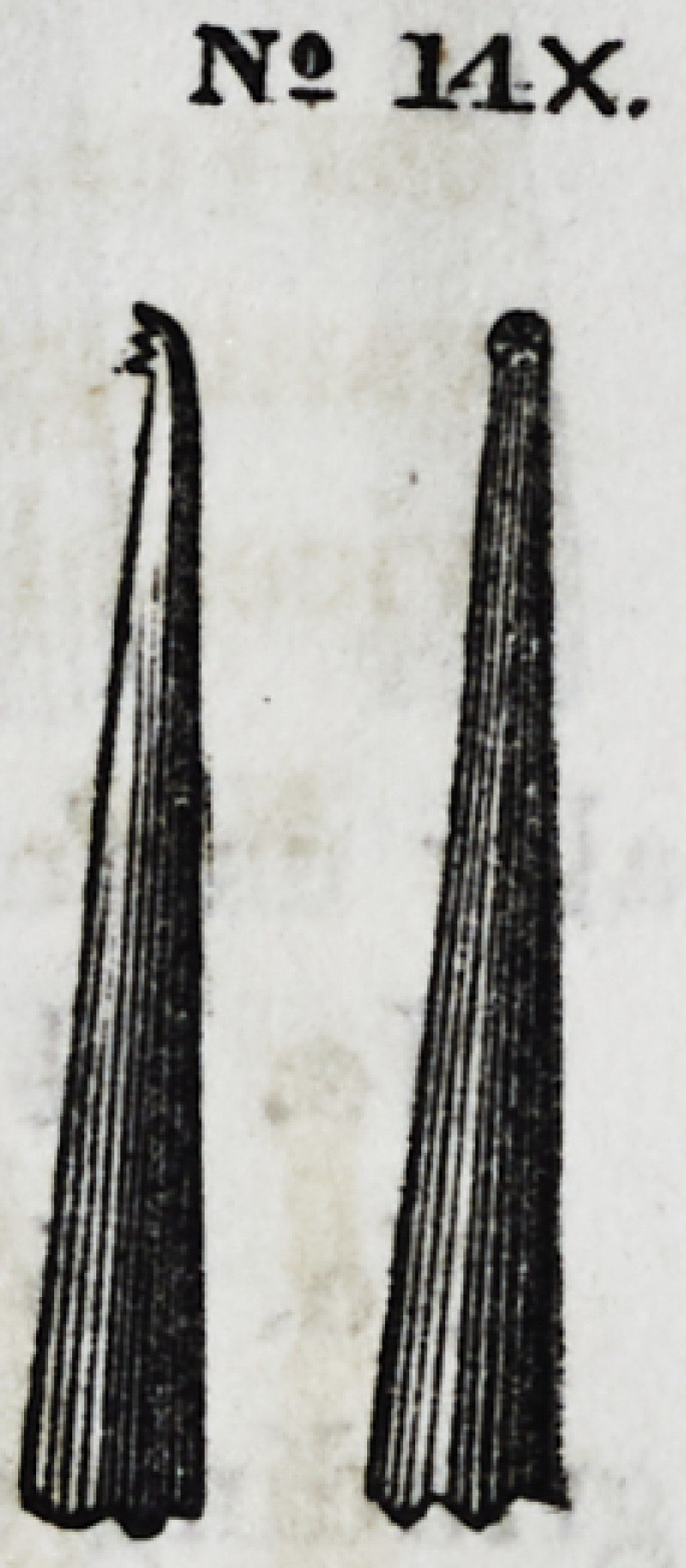


**No 15 f17:**
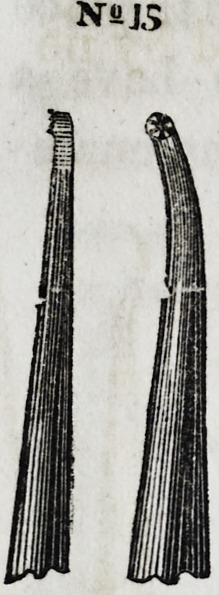


**No 16. f18:**
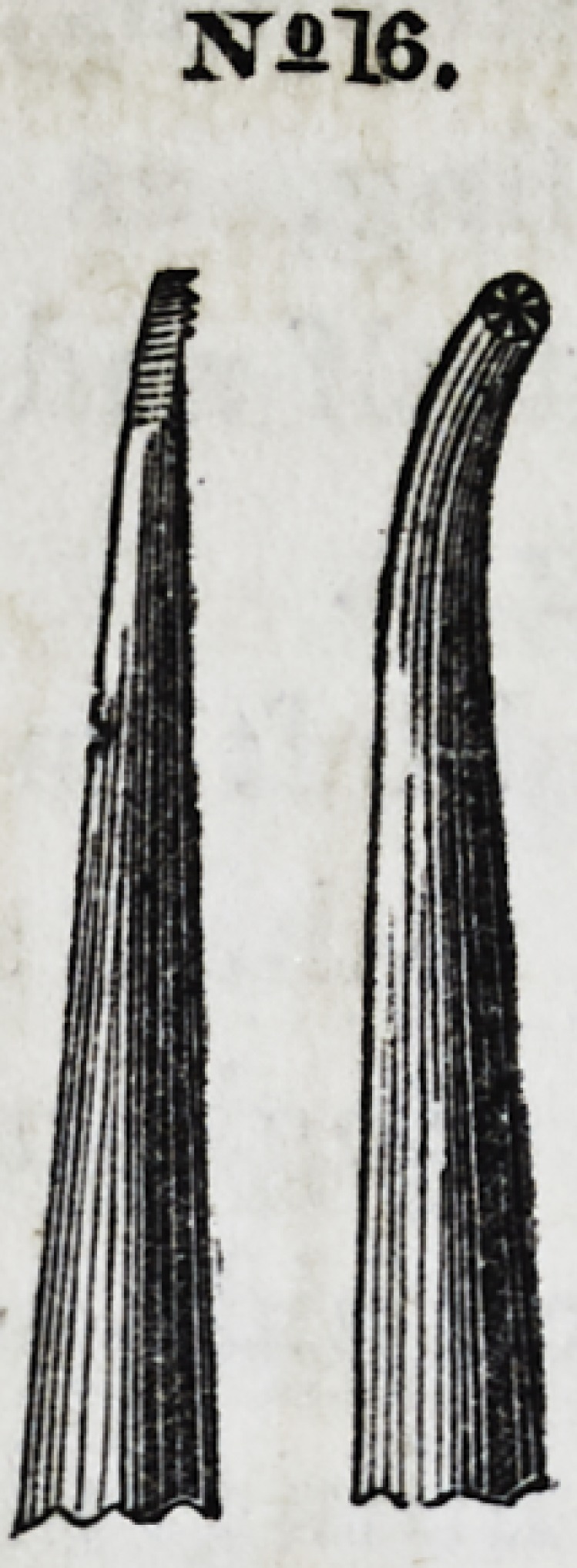


**No 17. f19:**
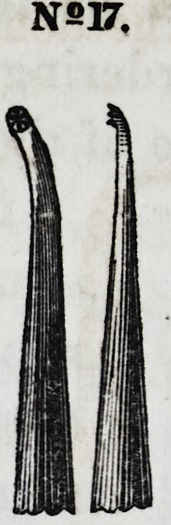


**No 18. f20:**
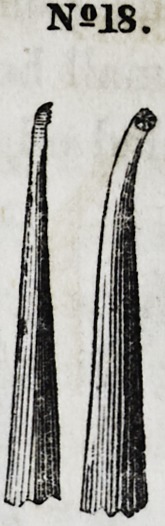


**No 19 f21:**
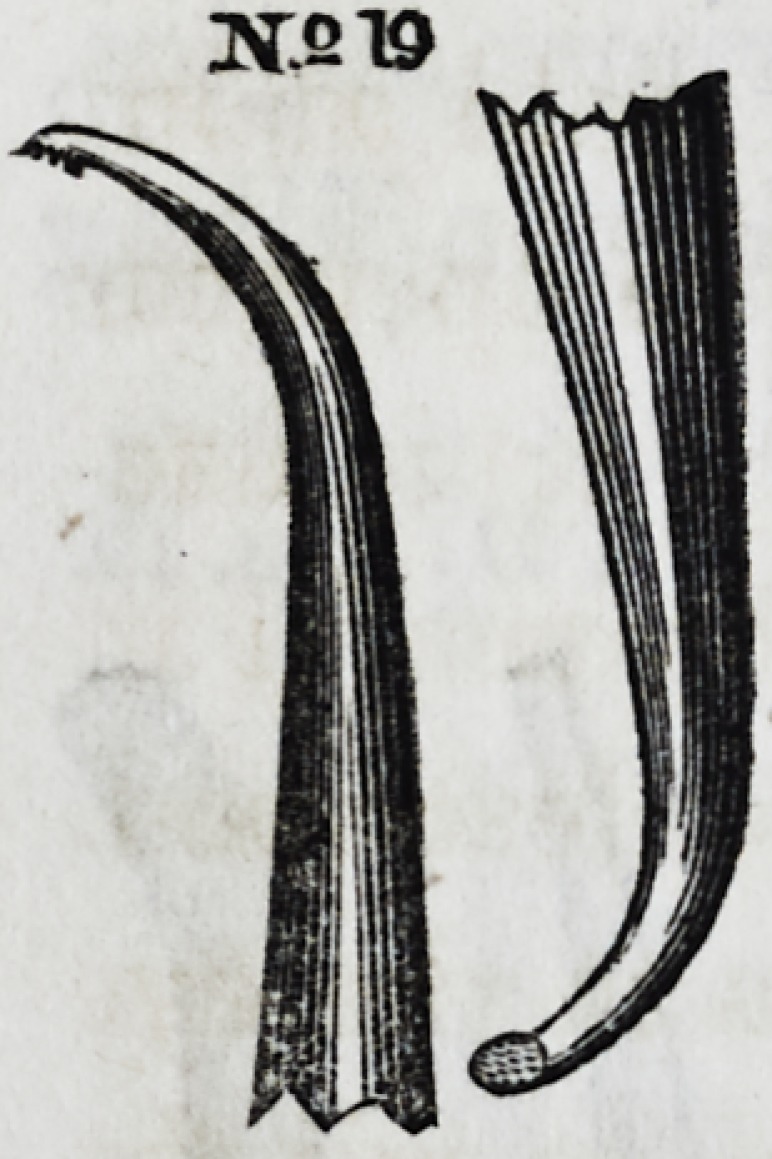


**No 20. f22:**
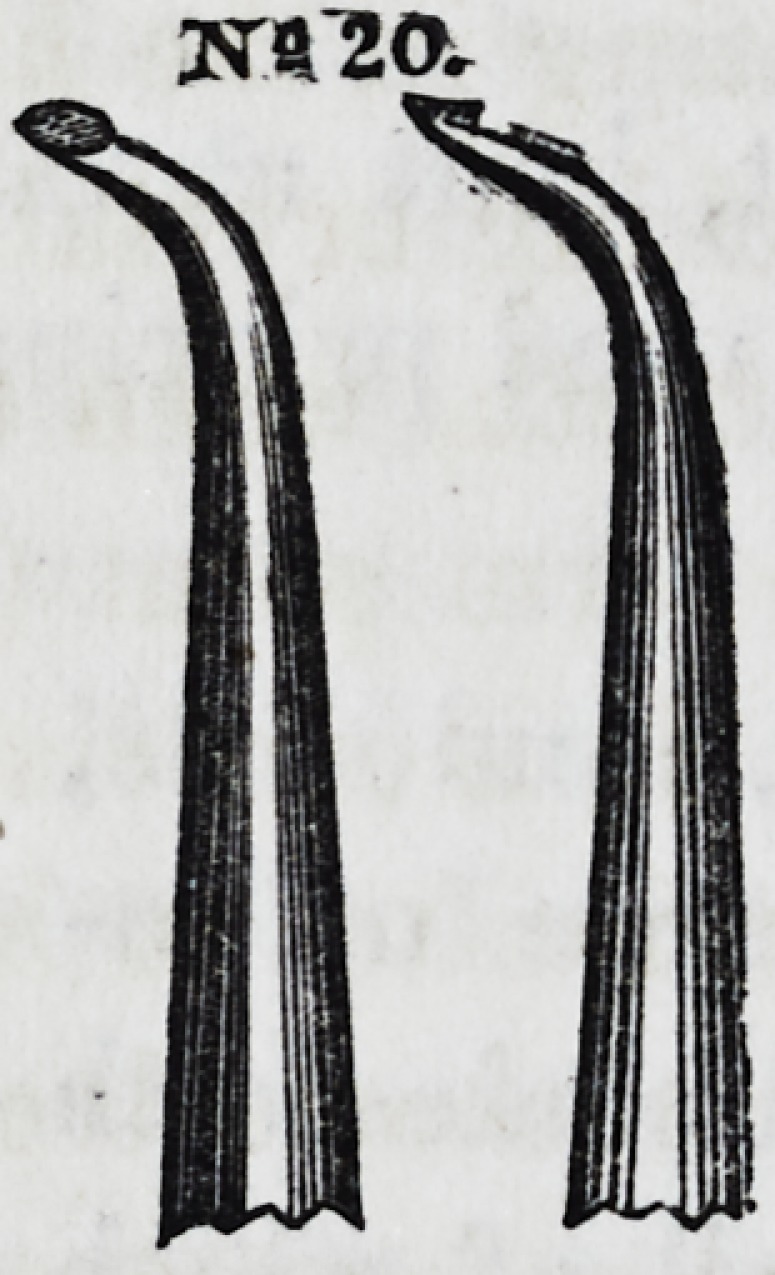


**No 21 f23:**
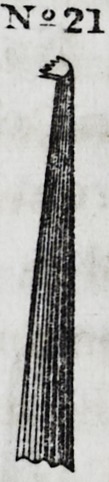


**No 22. f24:**
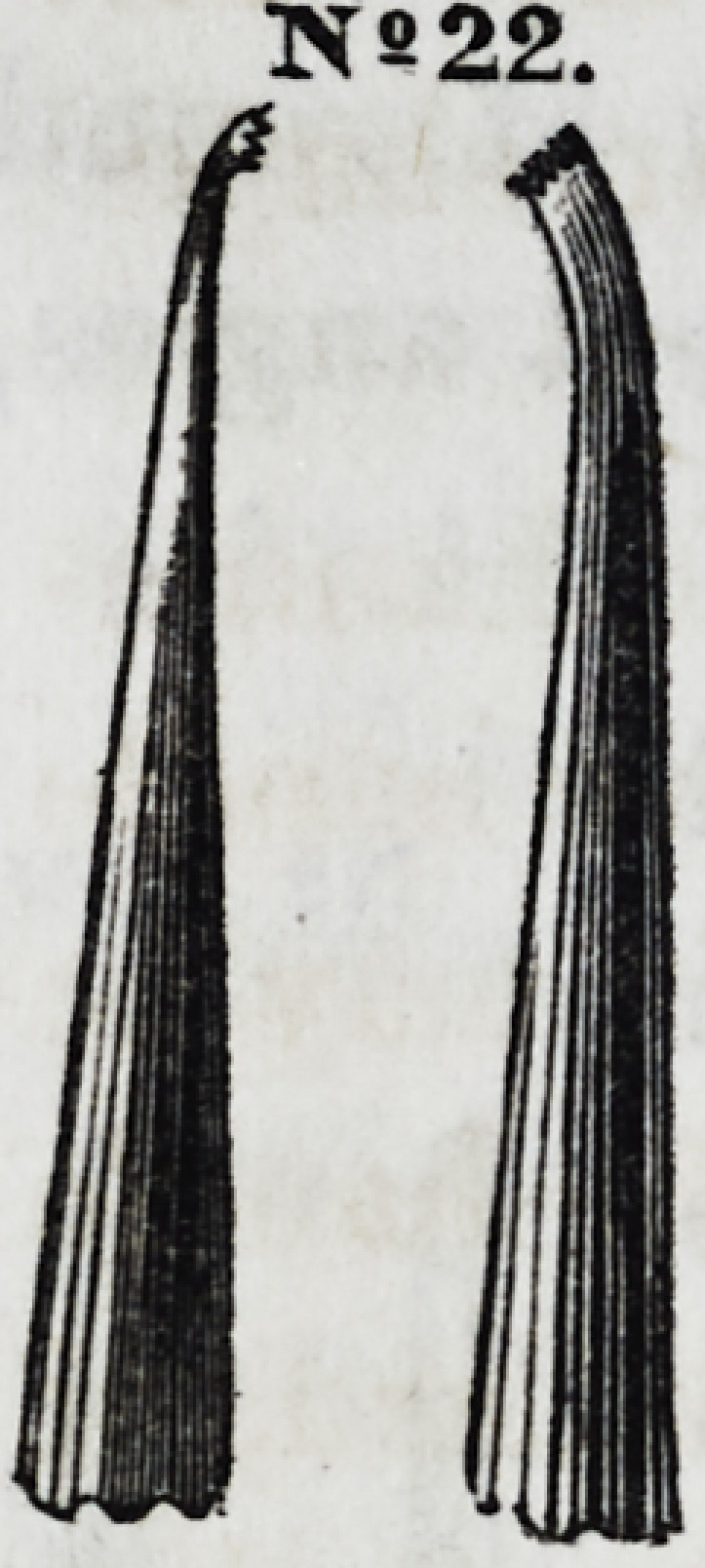


**No 23. f25:**
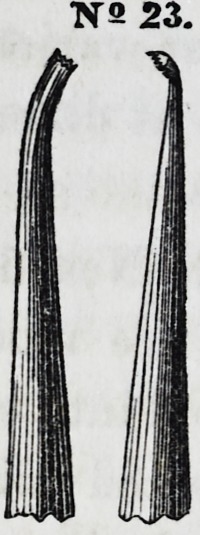


**No. 24. f26:**
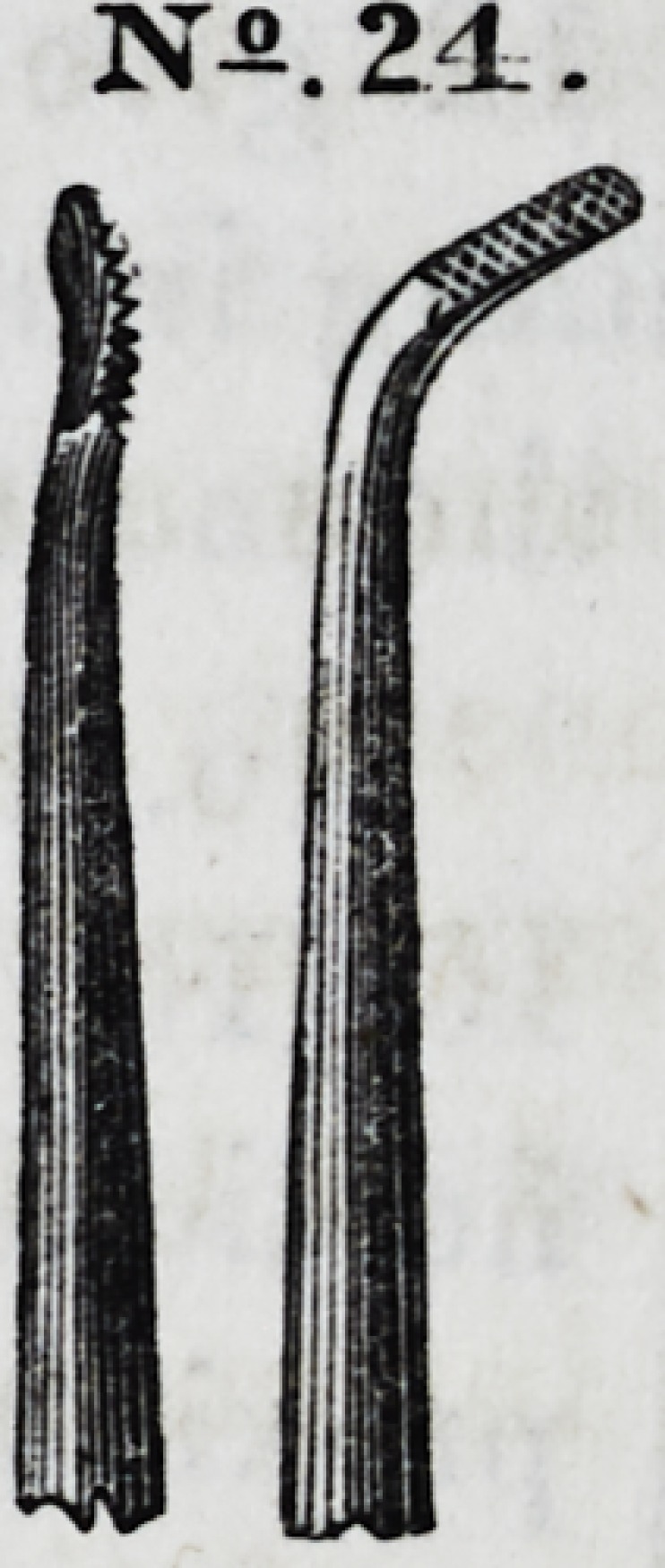


**No 25. f27:**
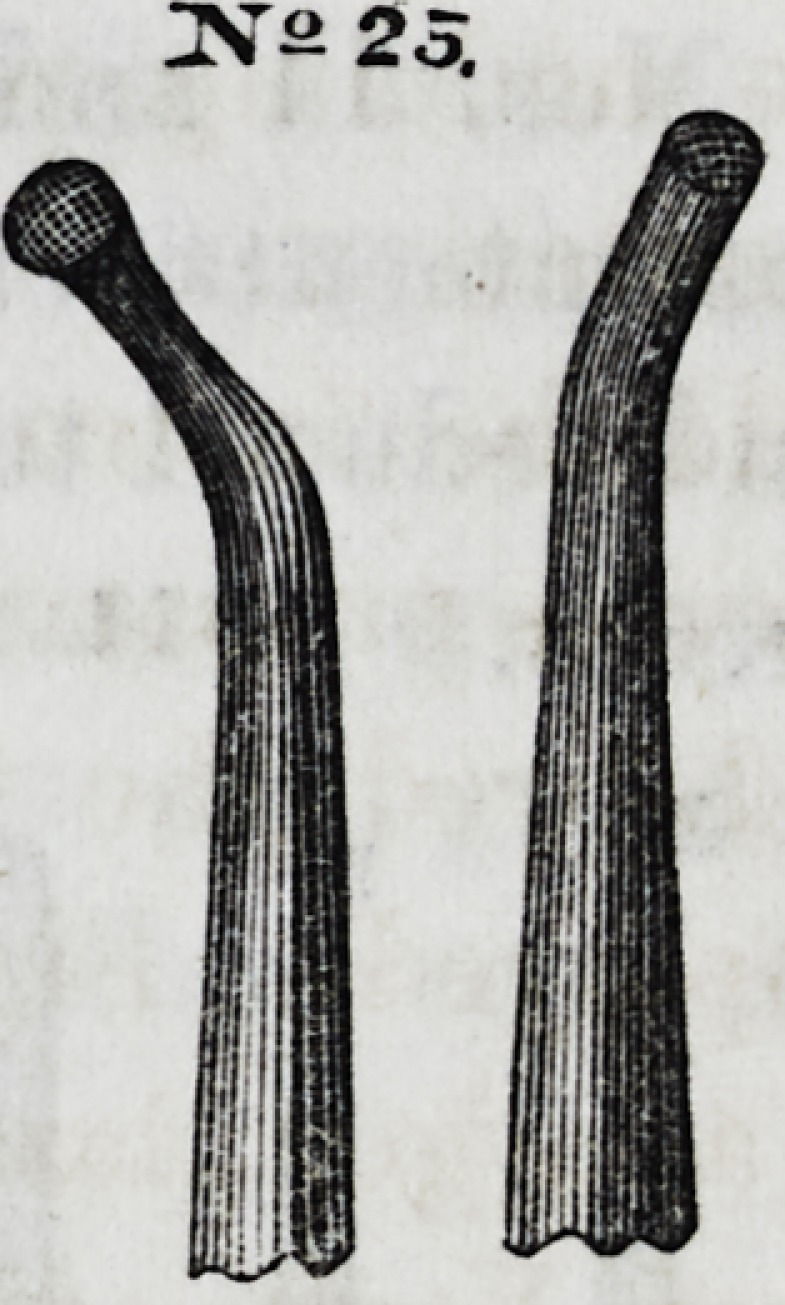


**No 26. f28:**
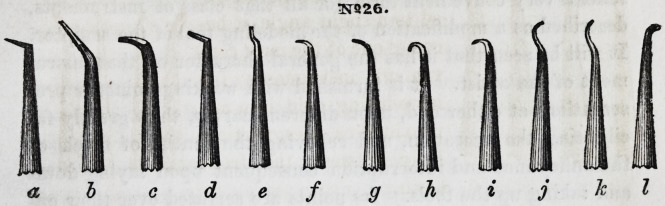


**FIG. A. f29:**
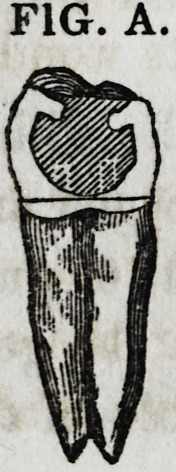


**FIG. B. f30:**
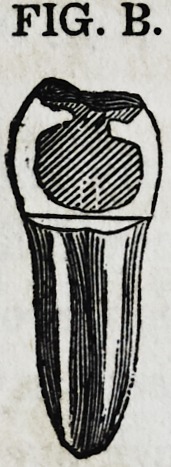


**FIG. C. f31:**
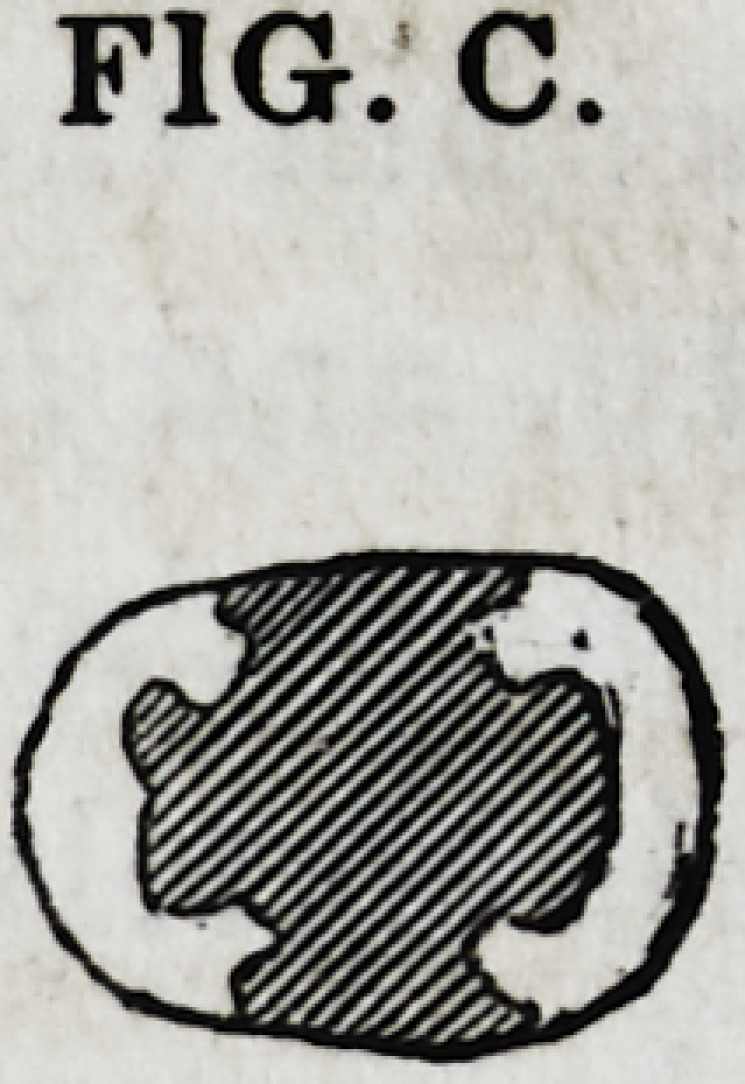


**FIG. D. f32:**
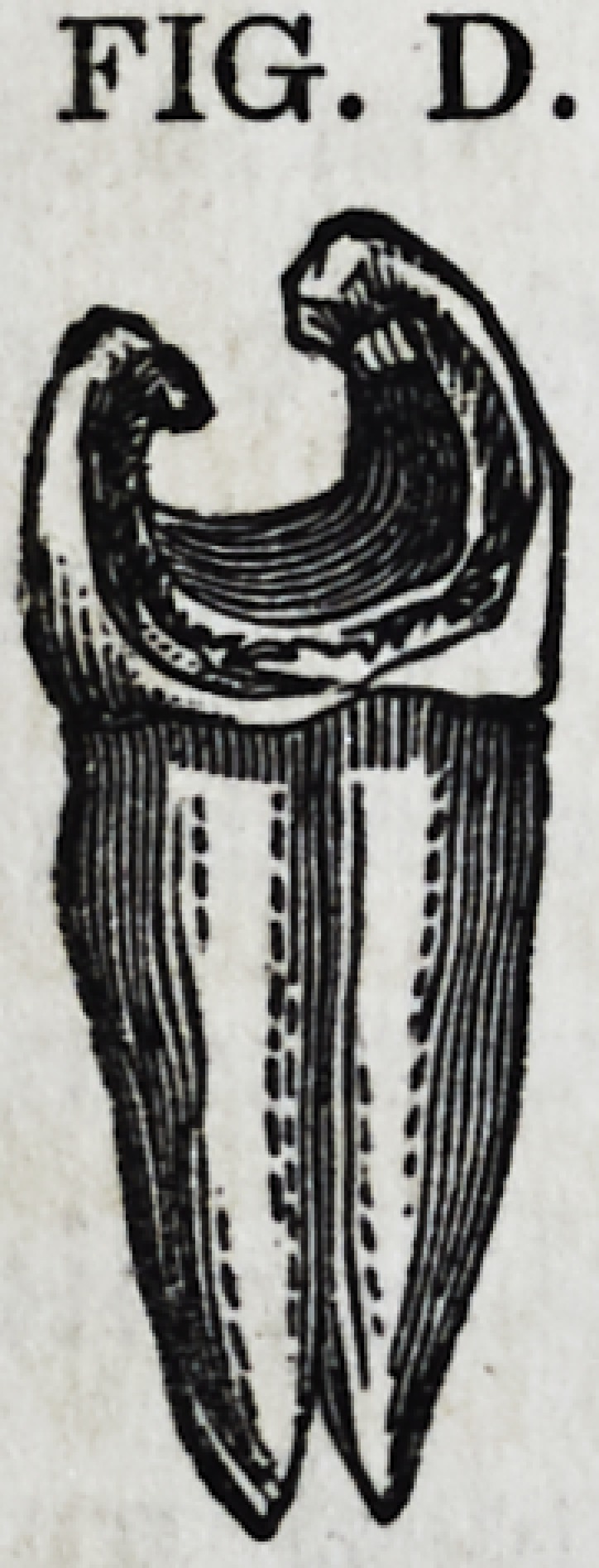


**FIG. E. f33:**
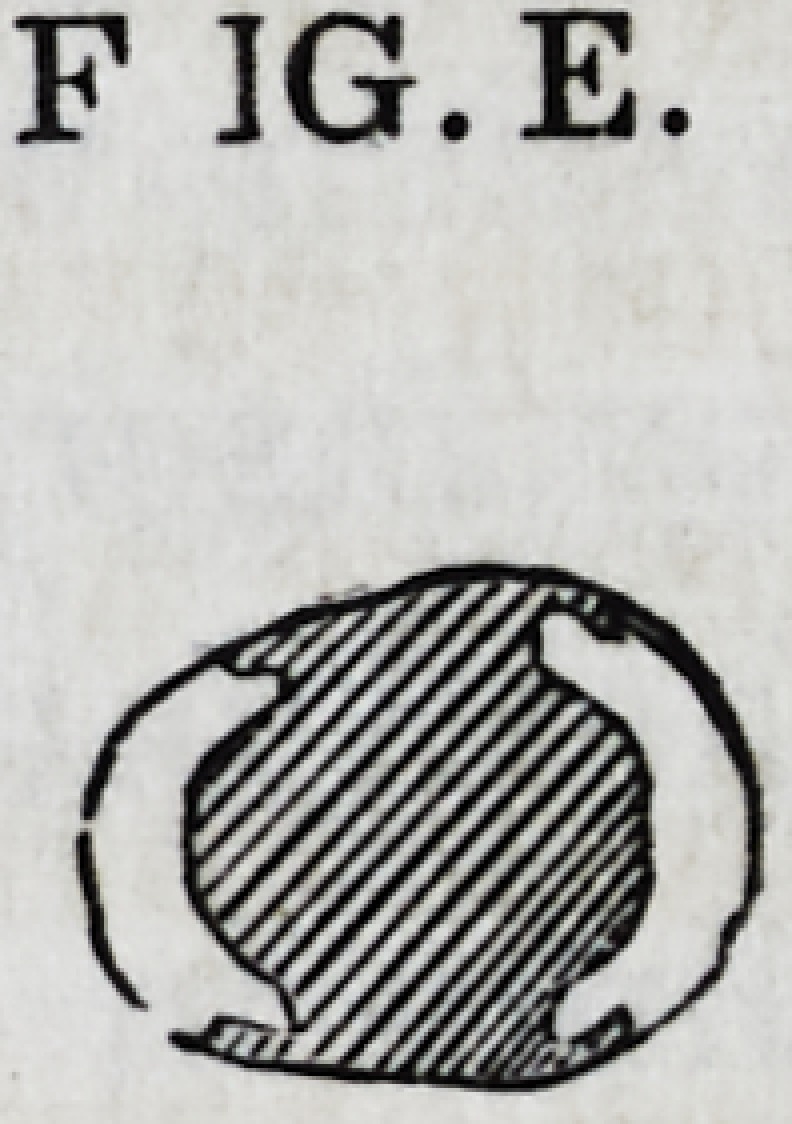


**FIG. F. f34:**
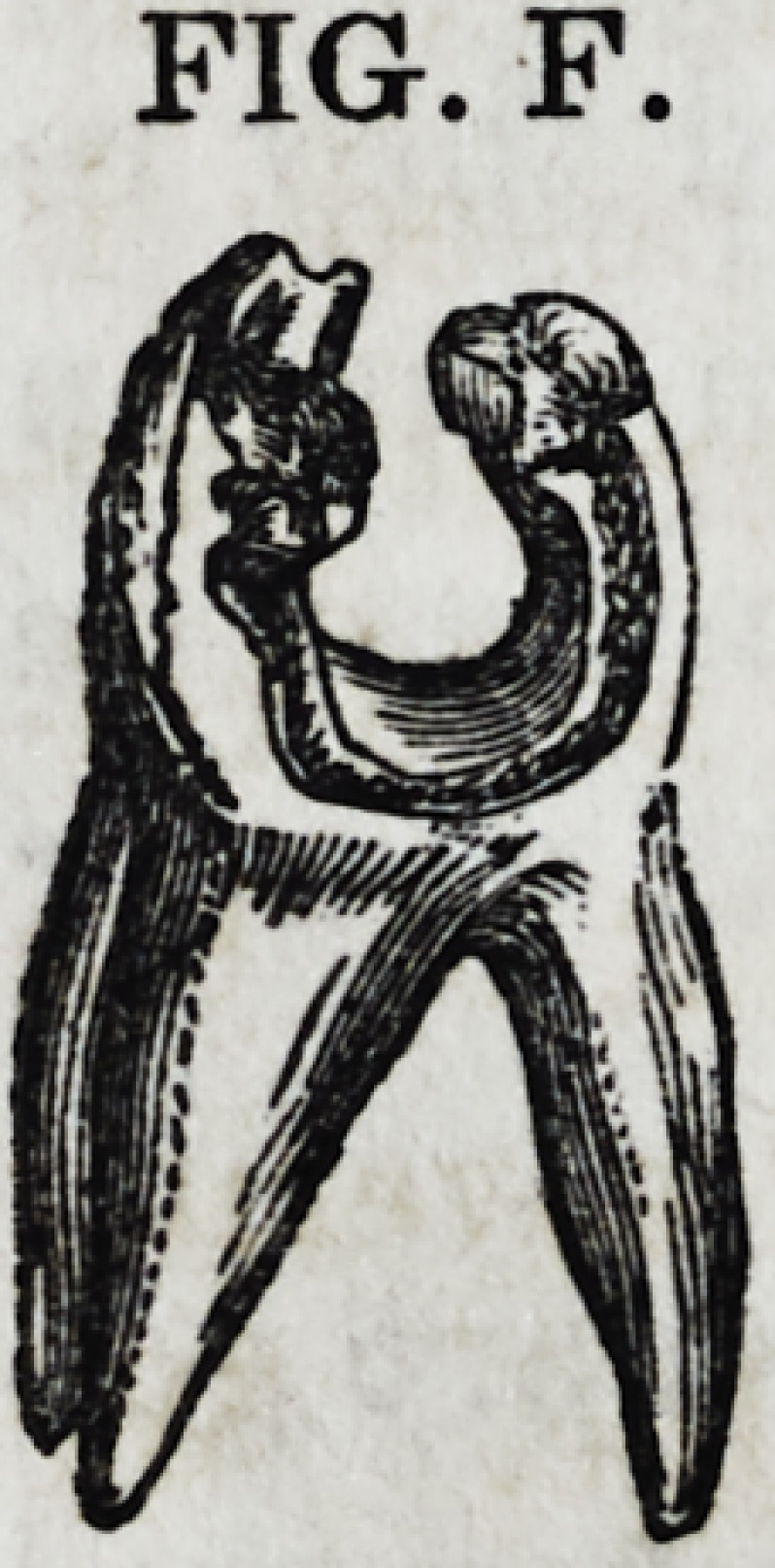


**FIG. G. f35:**
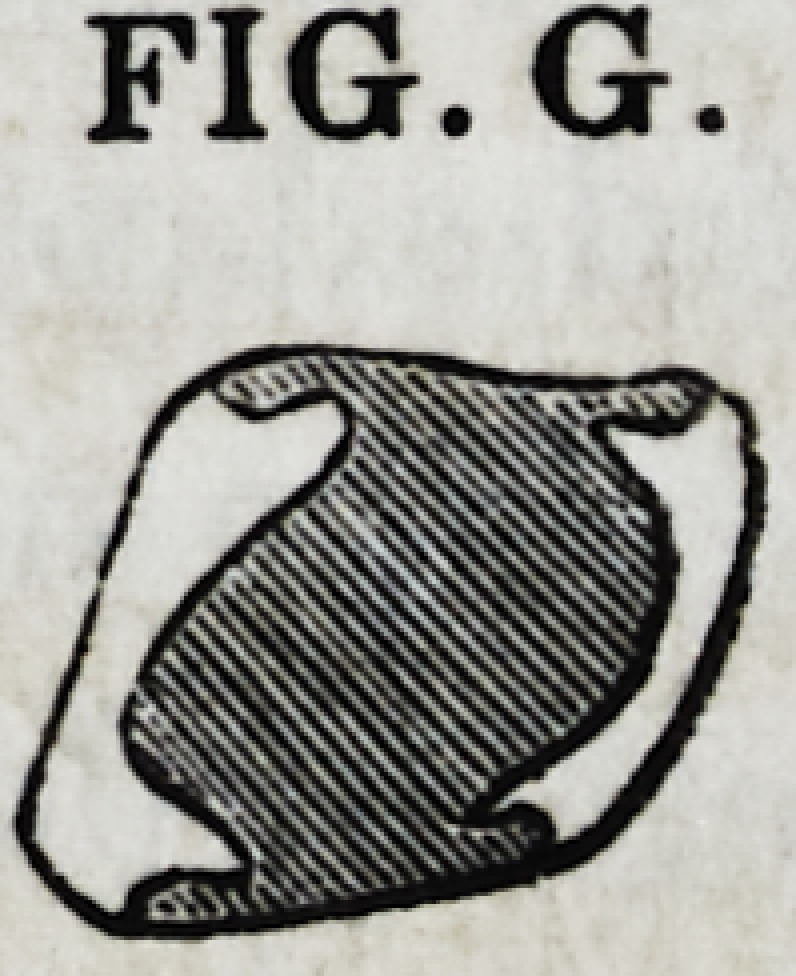


**FIG. H. f36:**
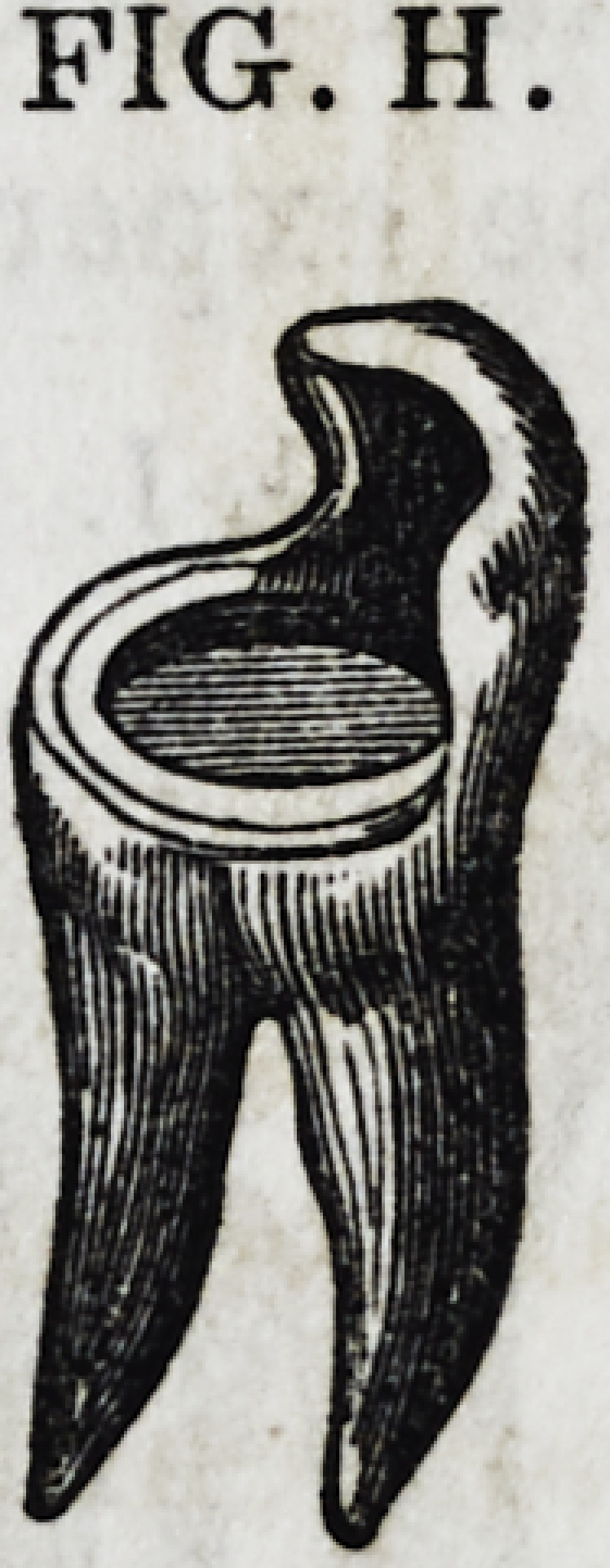


**FIG. I. f37:**
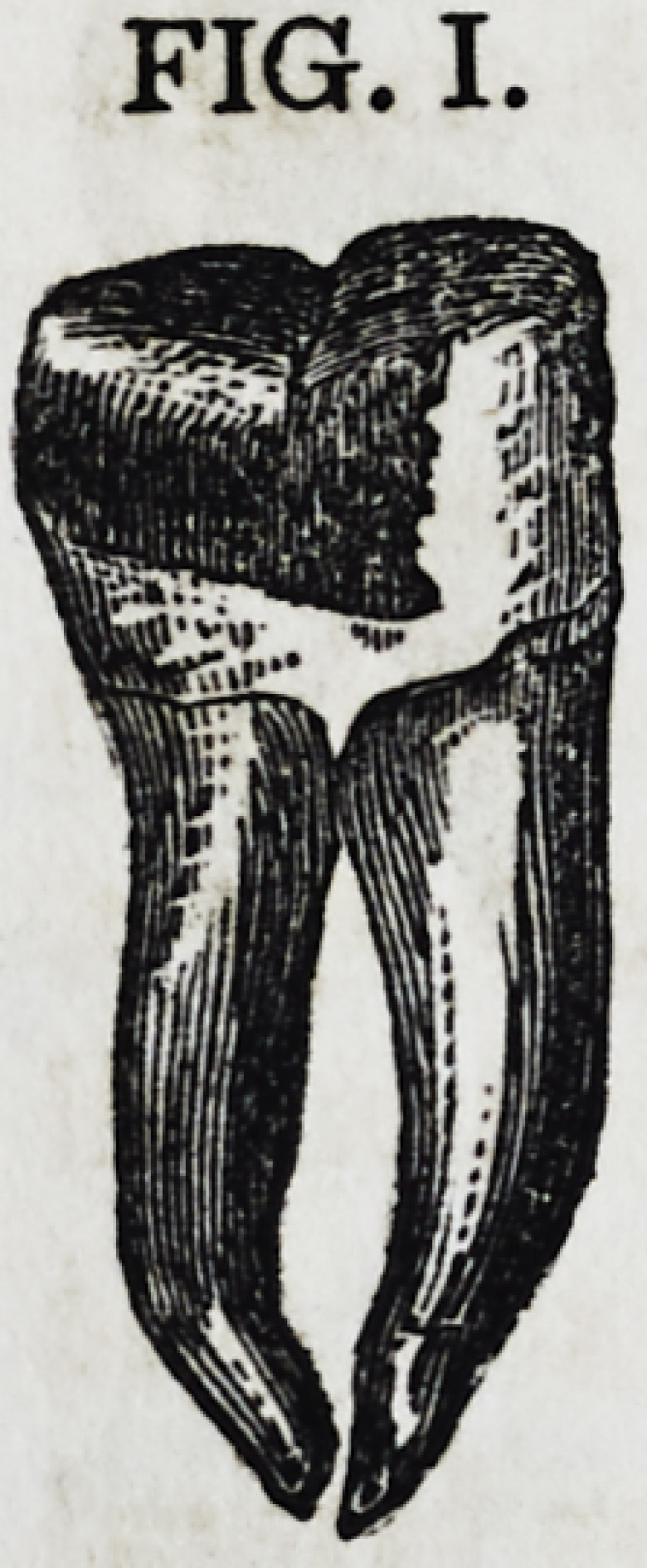


**FIG. J. f38:**
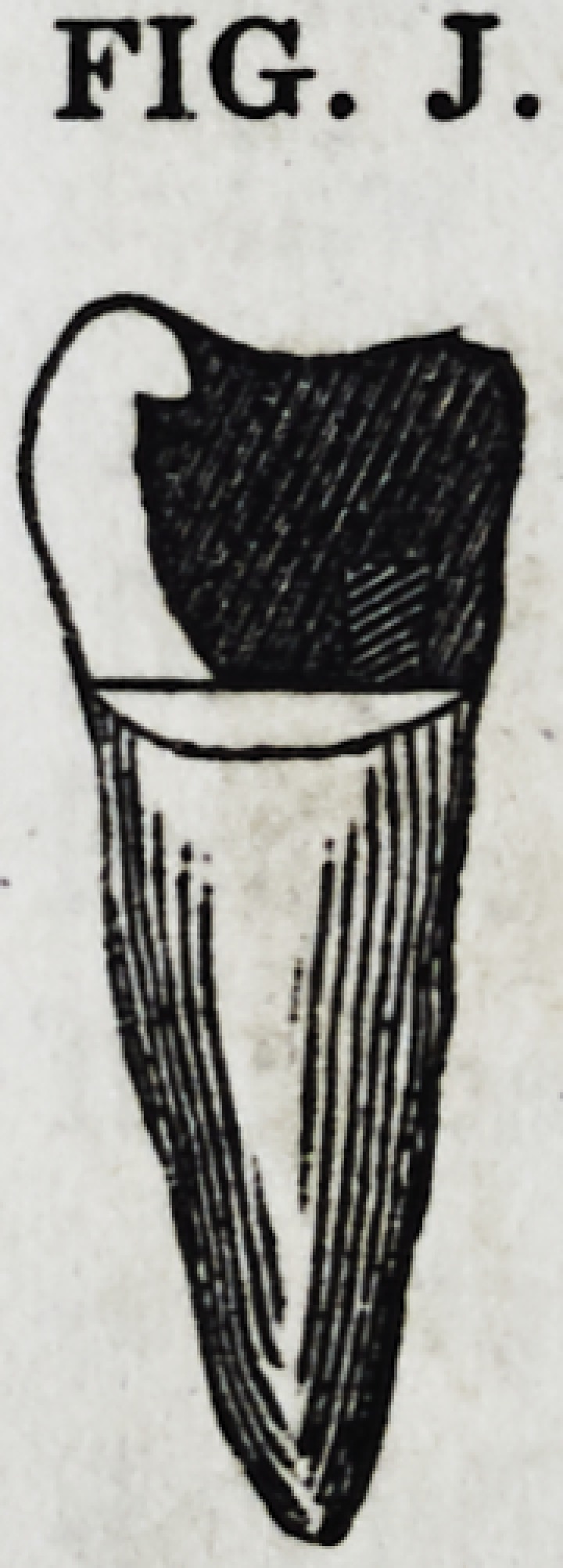


**FIG. K. f39:**
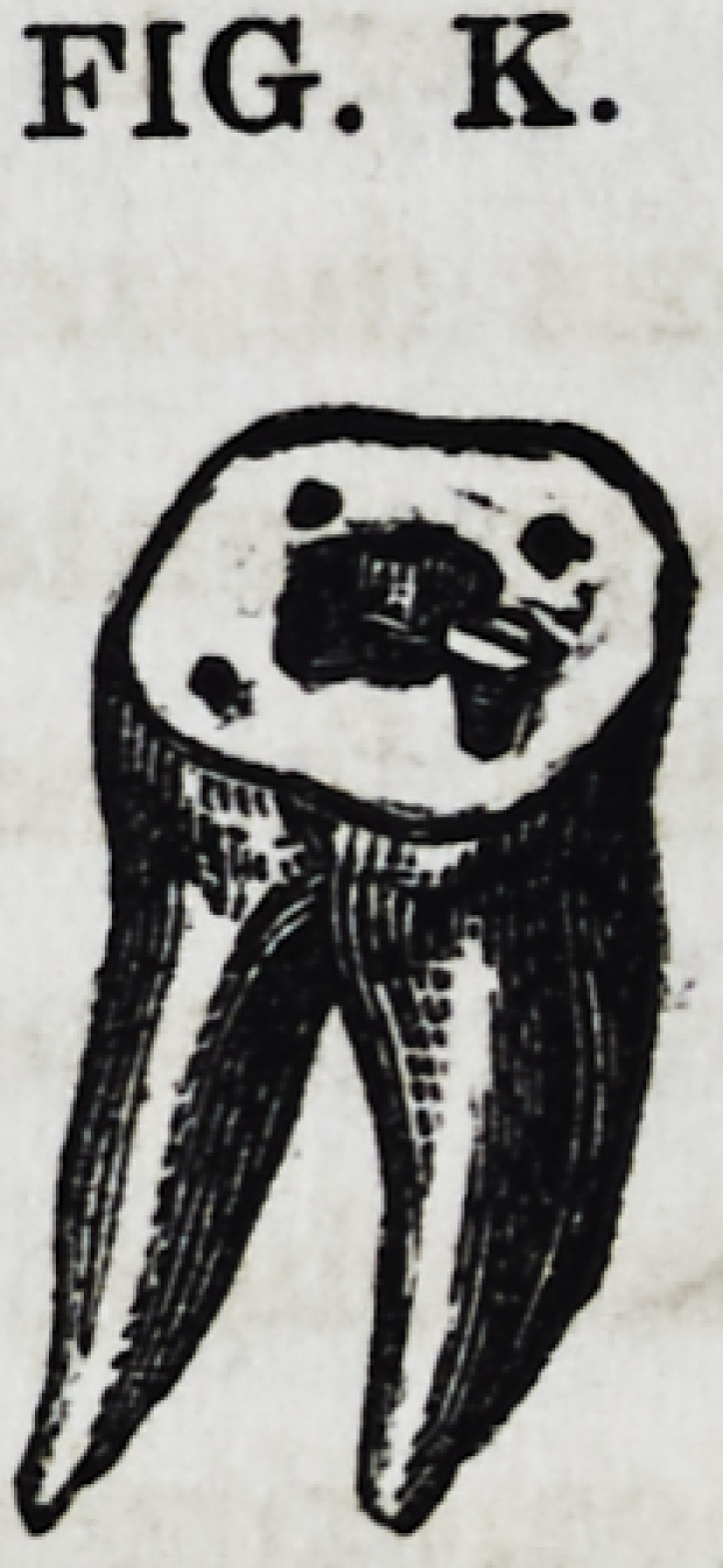


**FIG. L. f40:**
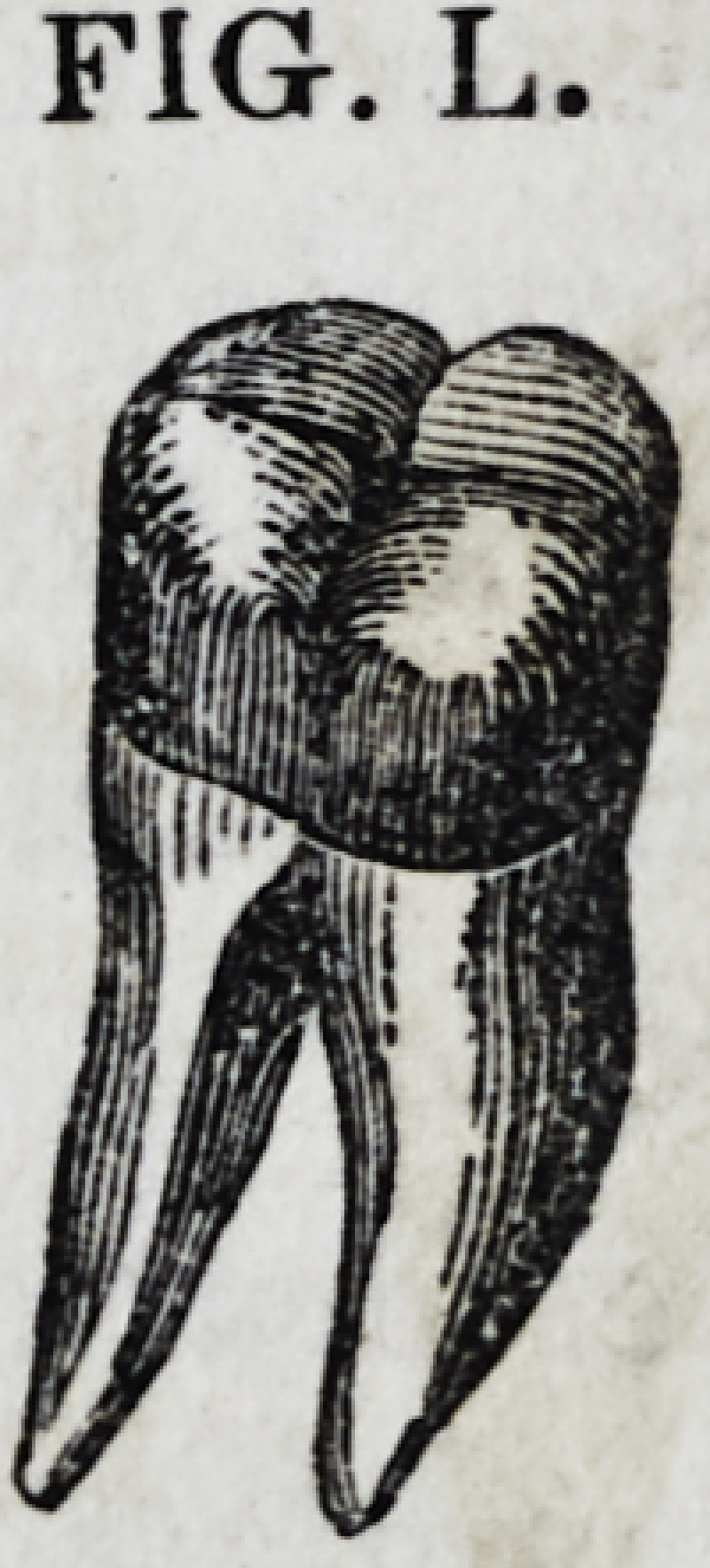


**FIG. M. f41:**
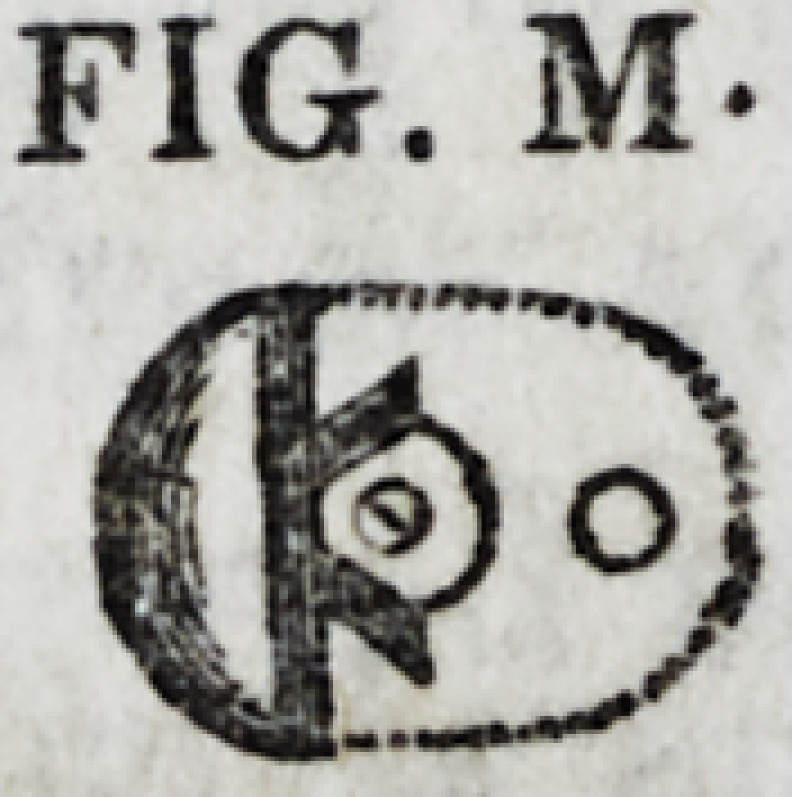


**FIGN. f42:**
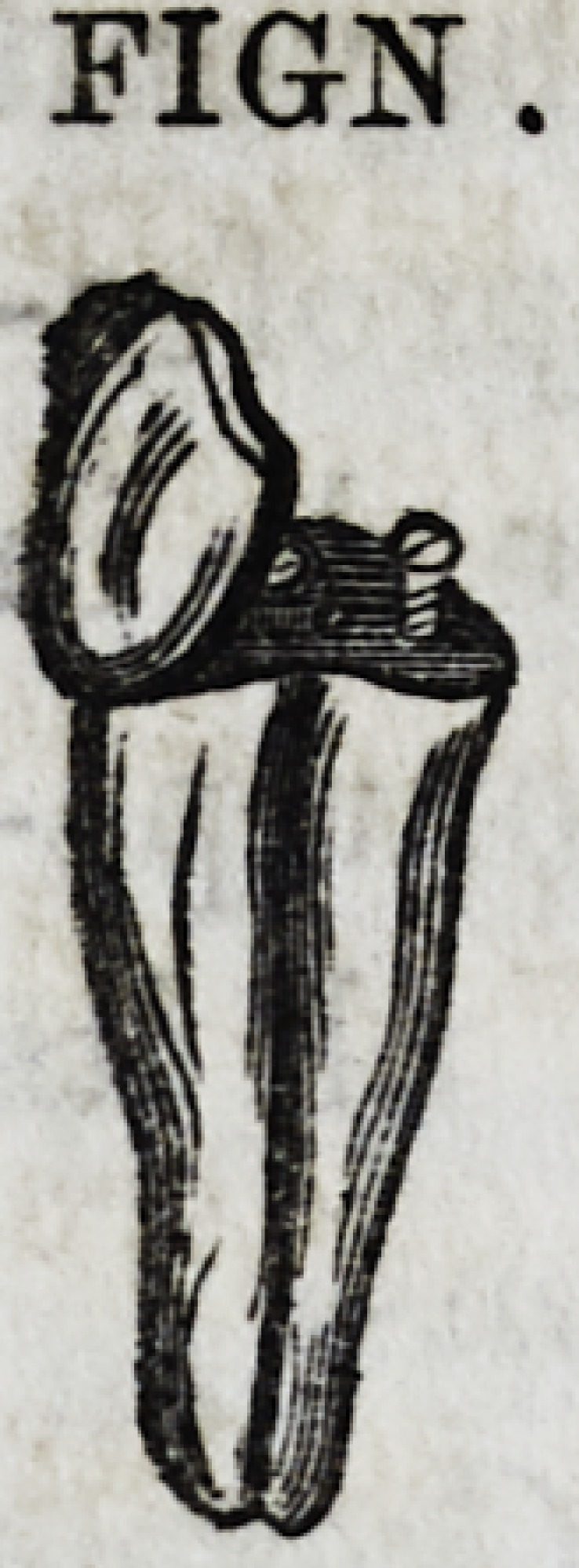


**FIG. Nx. f43:**
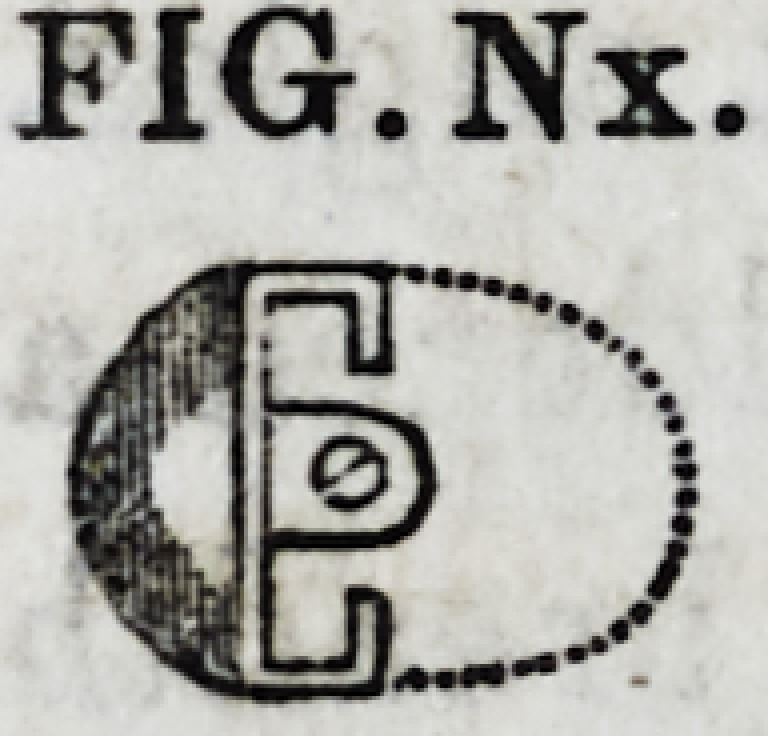


**FIG. O. f44:**
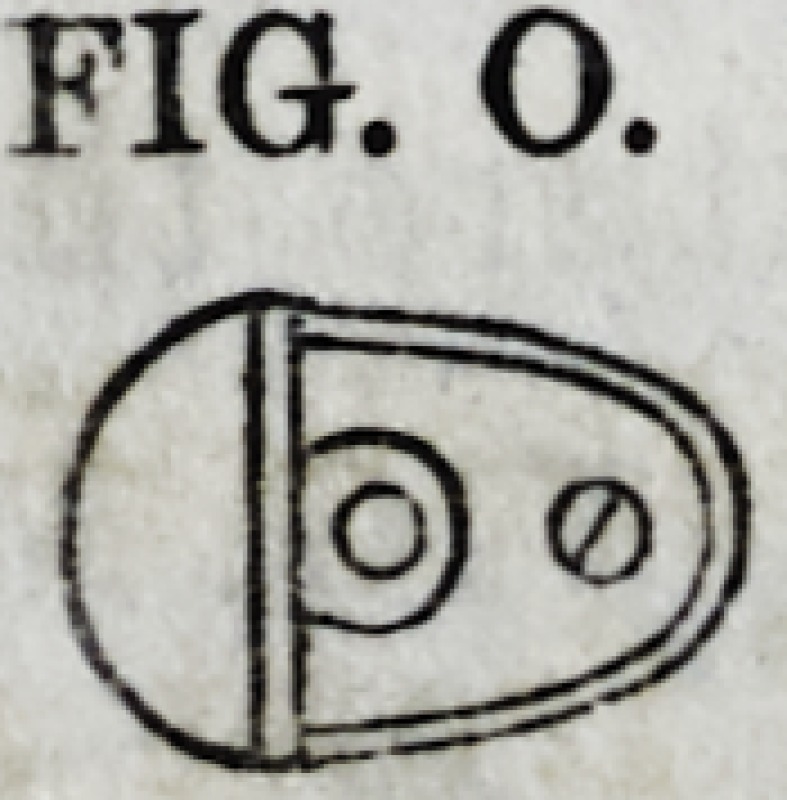


**FIG. P. f45:**
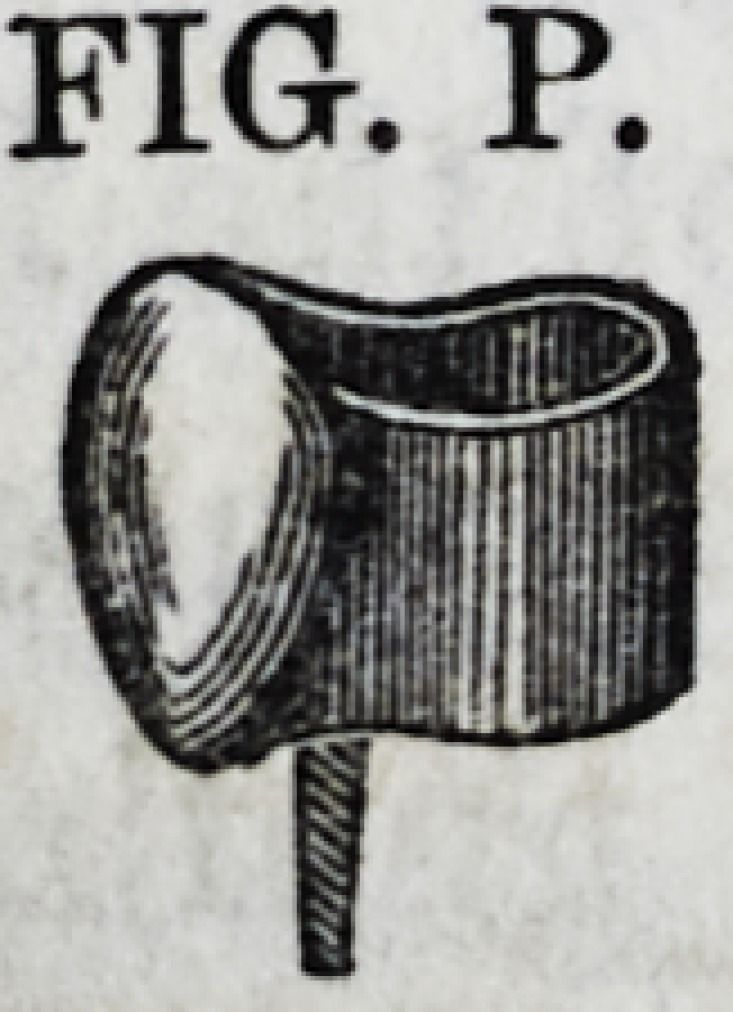


**FIG. Q. f46:**
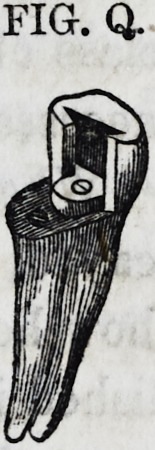


**FIG. R. f47:**
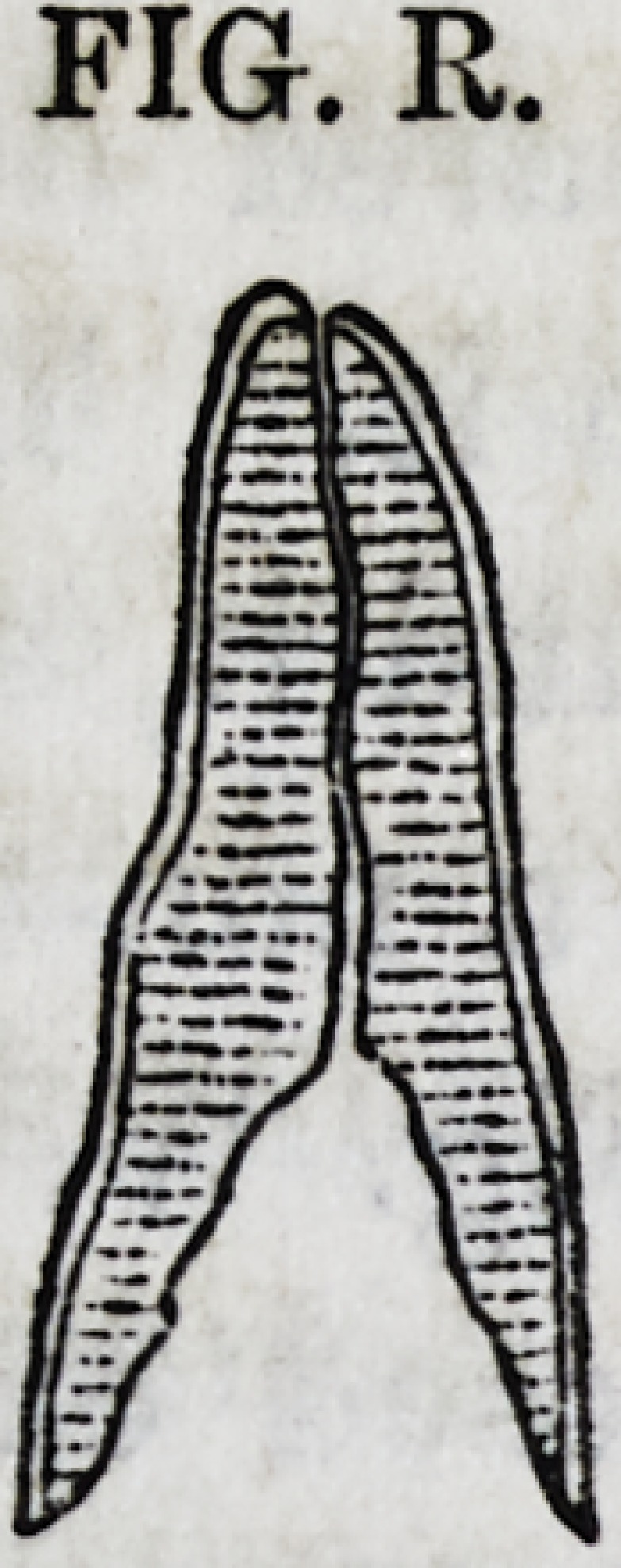


**FIG. S. f48:**
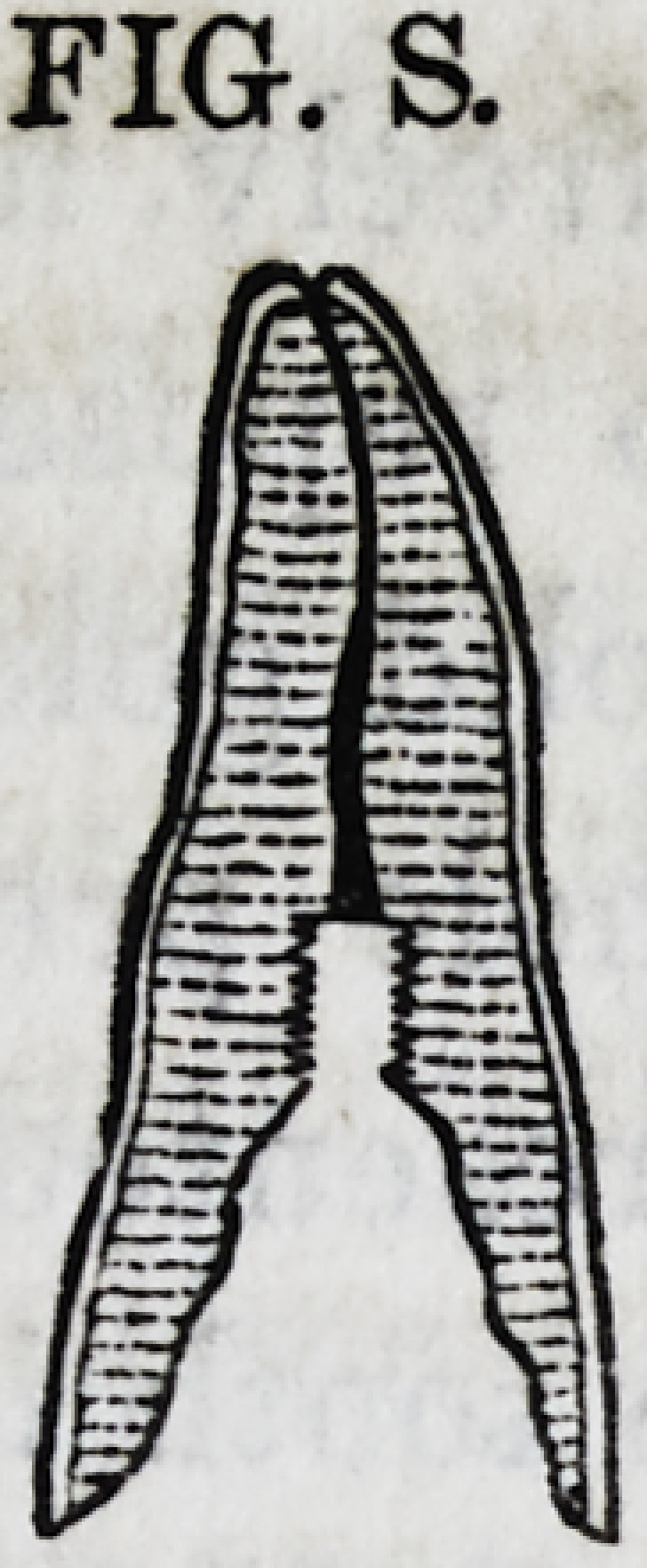


**T. f49:**
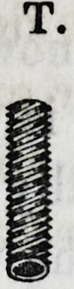


**U. f50:**
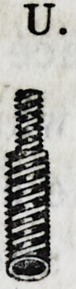


**FIG. V. f51:**
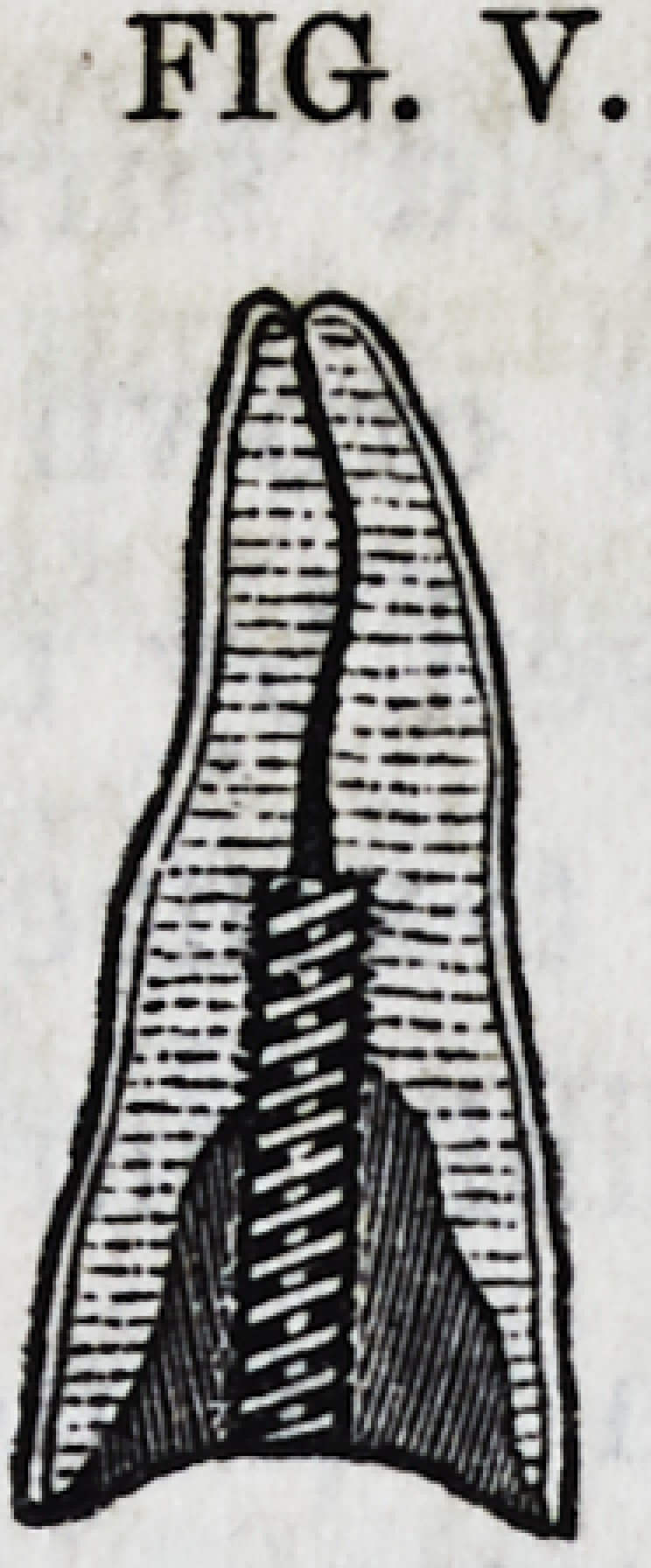


**FIG. W. f52:**
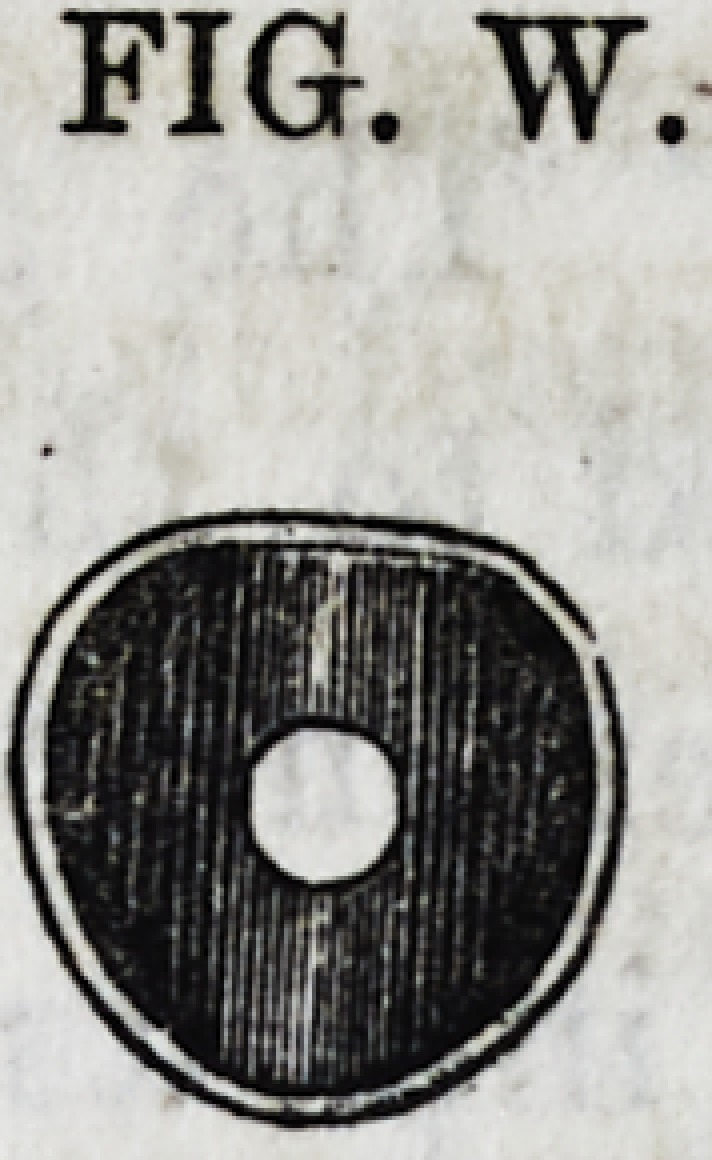


**FIG.1. f53:**
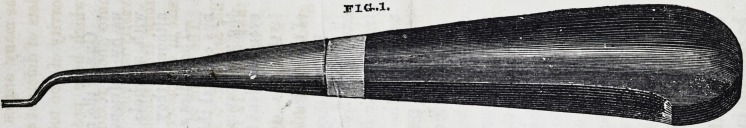


**FIG.2. f54:**
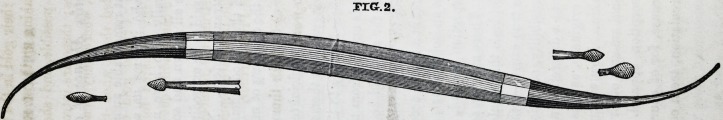


**FIG.3. f55:**
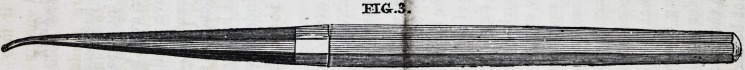


**FIG.4. f56:**